# Recent Progress on Non‐Carbon‐Supported Single‐Atom Catalysts for Electrochemical Conversion of Green Energy

**DOI:** 10.1002/smsc.202300010

**Published:** 2023-04-12

**Authors:** Yiming Zhu, Jingyao Wang, Jiwei Ma

**Affiliations:** ^1^ Shanghai Key Laboratory for R&D and Application of Metallic Functional Materials Institute of New Energy for Vehicles School of Materials Science and Engineering Tongji University Shanghai 201804 China

**Keywords:** green energy conversion, metal–support interactions, non-carbon supports, single-atom catalysts, synthesis methods

## Abstract

Single‐atom catalysts (SACs) are a popular area of research for clean energy conversion owing to their cost‐effectiveness and excellent performance. The support plays a vital role in uniformly stabilizing and dispersing the single atoms. Although easily accessible carbon (C) is commonly selected as a support for SACs, its electrochemical properties, particularly stability, usually limits its application. Recently, non‐C materials with flexible physicochemical properties and unique metal–support interactions have attracted increasing attention for loading isolated metal atoms, showing promise for promoting catalytic performance. Therefore, in this review, a comprehensive summary of current research developments in non‐C‐supported SACs for green energy conversion is provided. The review begins with a brief introduction of the four types of non‐C‐supported SACs based on the support used. Thereafter, a systemic summary of synthesis methods for non‐C‐supported SACs analyzing their advantages and disadvantages is provided. The interactions between single metal atoms and non‐C supports are discussed, followed by their applications in green energy conversion. Then, the significance of adopting a variety of in situ/operando approaches is emphasized to gain insight into both the synthesis and reaction mechanisms, which have been successfully deployed for non‐C‐supported SACs. Finally, the remaining challenges and perspectives on designing promising non‐C‐supported SACs are discussed.

## Graphical abstract

A promising electrocatalyst is critical for solving issues of resource scarcity and environment pollution. This review summarizes the latest research progress in non‐carbon‐supported single‐atom catalysts for green energy conversion, including support species, synthesis methods, metal–support interactions, and in situ/operando characterizations. By providing a systematic overview, this review can facilitate the rational design of advanced electrocatalysts and benefit researchers in the field.

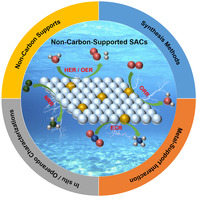

## 1 Introduction

Exponential growth in industrialization and urbanization has led to higher energy consumption. Presently, fossil fuels remain the primary source of energy. However, they are difficult to renew and have limited underground reserves. Furthermore, fossil fuels generate harmful exhaust gases upon combustion, adversely affecting the environment. Therefore, concerns regarding energy their scarcity and environmental pollution crises have been reported.^[^ ^1^, ^2^, ^3^ ^]^ Recently, there has been a surge in environmental awareness worldwide, with a growing emphasis on energy issues in numerous countries. The efficient transformation and utilization of green resources are becoming the main goals for future development.^[^ ^4^, ^5^, ^6^ ^]^ Electrochemical energy conversion, such as the oxygen reduction reaction (ORR), water splitting reaction, nitrogen reduction reaction (NRR), and electrochemical carbon dioxide reduction reaction (ECR), convert green feedstock into electricity or use electricity to convert simple small molecules into high‐value products. These conversion processes have attracted much attention due to the advantages of abundant reactants, nonpolluting emissions, and high energy efficiency.^[^ ^7^, ^8^, ^9^, ^10^, ^11^ ^]^ The desired electrocatalysts are indispensable for enhancing catalytic performance by accelerating the reaction rate and reducing the kinetic barrier.^[^ ^12^, ^13^, ^14^, ^15^ ^]^

Previously, researchers found that the size of an electrocatalyst significantly affects its catalytic activity. Ultrasmall nanoparticles and nanoclusters are usually more active than their bulk counterparts due to their large surface area and the high number of exposed atoms.^[^ ^16^, ^17^, ^18^, ^19^, ^20^ ^]^ However, with the rapid development of characterization tools such as high‐resolution electronic microscopy and synchrotron radiation techniques, the size of nanomaterials has been pushed even further to the microscopic limit, resulting in the development of single‐atom materials.^[^ ^21^, ^22^, ^23^, ^24^, ^25^ ^]^ The concept of single‐atom catalysts (SACs) was first mentioned in Zhang's pioneering work in 2011, which successfully designed isolated platinum (Pt) atoms supported on FeO_*x*_ for efficient carbon monoxide (CO) oxidation via strong electrostatic and covalent bonding interactions.^[^ ^26^ ^]^ Considerable efforts were invested in fabricating highly efficient SACs and conducting a thorough structural analyses of these materials.^[^ ^27^, ^28^, ^29^, ^30^, ^31^, ^32^ ^]^ Multiple researchers have confirmed that SACs exhibit superior activity due to their maximized atom utilization, abundant unsaturated coordination configurations, and unique quantum size effects. In addition, the minimal amount of metal precursors required for synthesizing SACs ensures a significant cost reduction. Because SACs with well‐defined active sites have a more elementary structure than conventional heterogeneous catalysts, they can serve as ideal model catalysts to establish the relationship between the structure and the resulting performance, providing both experimental and theoretical guidance for the rational fabrication of promising catalysts.^[^ ^33^, ^34^, ^35^, ^36^, ^37^, ^38^, ^39^, ^40^, ^41^, ^42^ ^]^

It is widely recognized that the surface free energy of nanomaterials increases significantly as their size decreases, leading to the aggregation of dispersed atoms.^[^ ^43^, ^44^, ^45^ ^]^ Therefore, a solid support is essential for thermally stable SACs that can provide available anchor sites for metal atoms to remain isolated during preparation and reaction processes. In addition, suitable supports with the ability to induce efficient metal–support interactions are critical for regulating catalytic activity, stability, and selectivity.^[^ ^46^, ^47^, ^48^, ^49^ ^]^ Considering these factors, the choice of support is a pivotal prerequisite for constructing effective SACs. Various carbon (C) materials, including porous carbon black, graphene, graphdiyne, and derivatives of metal–organic frameworks (MOFs), have been popular as substrates for single atoms. These supports offer several advantages, including easy access and inexpensive raw materials. Additionally, the unique structure of C provides a large surface area, enhancing the stabilization of more single metal atoms.^[^ ^50^, ^51^, ^52^, ^53^ ^]^ Consequently, the majority of current SACs are incorporated into C supports, with their structures, catalytic performances, synthesis strategies, and reaction mechanisms well characterized and investigated. Correspondingly, several related reviews have been published on this topic.^[^ ^54^, ^55^, ^56^, ^57^, ^58^ ^]^ However, C tends to dissolve under electrochemical conditions, resulting in unstable C‐supported SACs. In addition, the simplicity of C limits the variety of coordination environments and metal–support interactions.^[^ ^59^, ^60^, ^61^ ^]^ As a result, more scientists have focused on using non‐C materials to atomically disperse metal atoms. For example, both cation and anion defects can be easily constructed on most non‐C supports to stabilize single metal atoms, and different defects render SACs with diverse catalytic properties. There is a wide variety of non‐C supports with flexible compositions and structures, in contrast to carbon, which can regulate the coordination environments and local charge states of single metal atoms.^[^ ^62^, ^63^, ^64^, ^65^, ^66^ ^]^ Accordingly, various and abundant metal–support interactions, such as electron redistribution, occur on non‐C‐supported SACs that are essential in boosting the activity by expediting the electronic conductivity and optimizing the energy barrier. The structures of non‐C supports are relatively more stable under harsh operating conditions, such as corrosive electrolyte and redox voltages. As a result, non‐C‐supported SACs usually exhibit improved catalytic durability.^[^ ^67^, ^68^, ^69^ ^]^ In addition, catalytic mechanism exploration has long been challenging for scientists. Ex situ technologies only provide structural, morphological, and compositional properties of catalysts, whereas in situ/operando characterizations can provide similar information under applied voltages. This discrepancy indicates that in situ/operando tools can monitor dynamic changes during the reaction process. These advanced characterizations can be further employed on non‐C‐supported SACs with simple and well‐defined structures that provide valuable opportunities to distinguish active sites, disclose structure–activity relationships, and propose highly accurate reaction mechanisms.

In the last few years, non‐C‐supported SACs have shown great promise in green energy conversion. Therefore, a timely comprehensive review is needed to summarize the research progress and provide guidance for future studies. The current review begins by summarizing and classifying four types of non‐C supports for SACs. Subsequently, it introduces synthesis strategies for preparing these SACs, analyzing their advantages and limitations. Additionally, we explore efficient metal–support interactions, including electron redistribution, covalent bonding, and synergistic functions, which can provide deep insights into the relationship between catalytic behaviors and structures. Finally, we demonstrate the comprehensive applications of non‐C‐supported SACs in typical green energy conversion, including the hydrogen evolution reaction (HER), oxygen evolution reaction (OER), ORR, ECR, and NRR. This review emphasizes the significance of in situ/operando characterization techniques for investigating the synthetic process and elucidates the reaction mechanism of non‐C‐supported SACs, highlighting their successful application. Finally, we discuss the remaining challenges and alternate perspectives in investigating non‐C‐supported SACs to enable more efficient green energy conversion. We believe the current review will provide new insights for researchers and inspire further interest in exploring non‐C‐supported SACs, thereby driving innovation in this emerging research field.

## 2 Non‐C Supports for SACs

As a representative heterogeneous catalyst, the solid supports of SACs are significant for the stability of single atoms and the introduction of efficient metal–support interactions.^[^ ^70^ ^]^ In this section, we classify various non‐C supports according to their species and then present their research progress.

### 2.1 Metals

Pure metals are typically utilized as catalysts in electrochemical reactions because of their suitable electronic properties. For instance, ruthenium (Ru) and iridium (Ir) have been used in water electrolyzers to generate hydrogen (H_2_), while Pt is commonly used in fuel cells.^[^ ^71^, ^72^, ^73^ ^]^ Adopting metals as supports for loading single metal atoms, known as single‐atom alloys (SAAs), may simultaneously take advantage of the host metal function and single‐atom effect.^[^ ^74^, ^75^, ^76^, ^77^ ^]^ Kyriakou et al. designed a new catalyst by employing a pure copper (Cu) substrate to support single palladium (Pd) atoms, and a novel concept of SAAs was proposed for the first time.^[^ ^78^, ^79^ ^]^ SAAs are more robust and stable in electrochemical reactions owing to the formation of strong metal–metal bonds. Moreover, the electronic structures of single metal atoms can be finely tuned by imposing an alloying strategy on metal supports that result in variable binding energies between the active atoms and intermediates for orienting specific reactions. Furthermore, the catalytic mechanism of SAAs can be easily discovered due to their simple and well‐defined structures, providing a theoretical guideline for rationally designing efficient catalysts.^[^ ^80^, ^81^, ^82^, ^83^, ^84^ ^]^ Therefore, SAAs have received considerable attention as promising catalytic systems with excellent properties for various green energy conversion.

**Figure 1:**
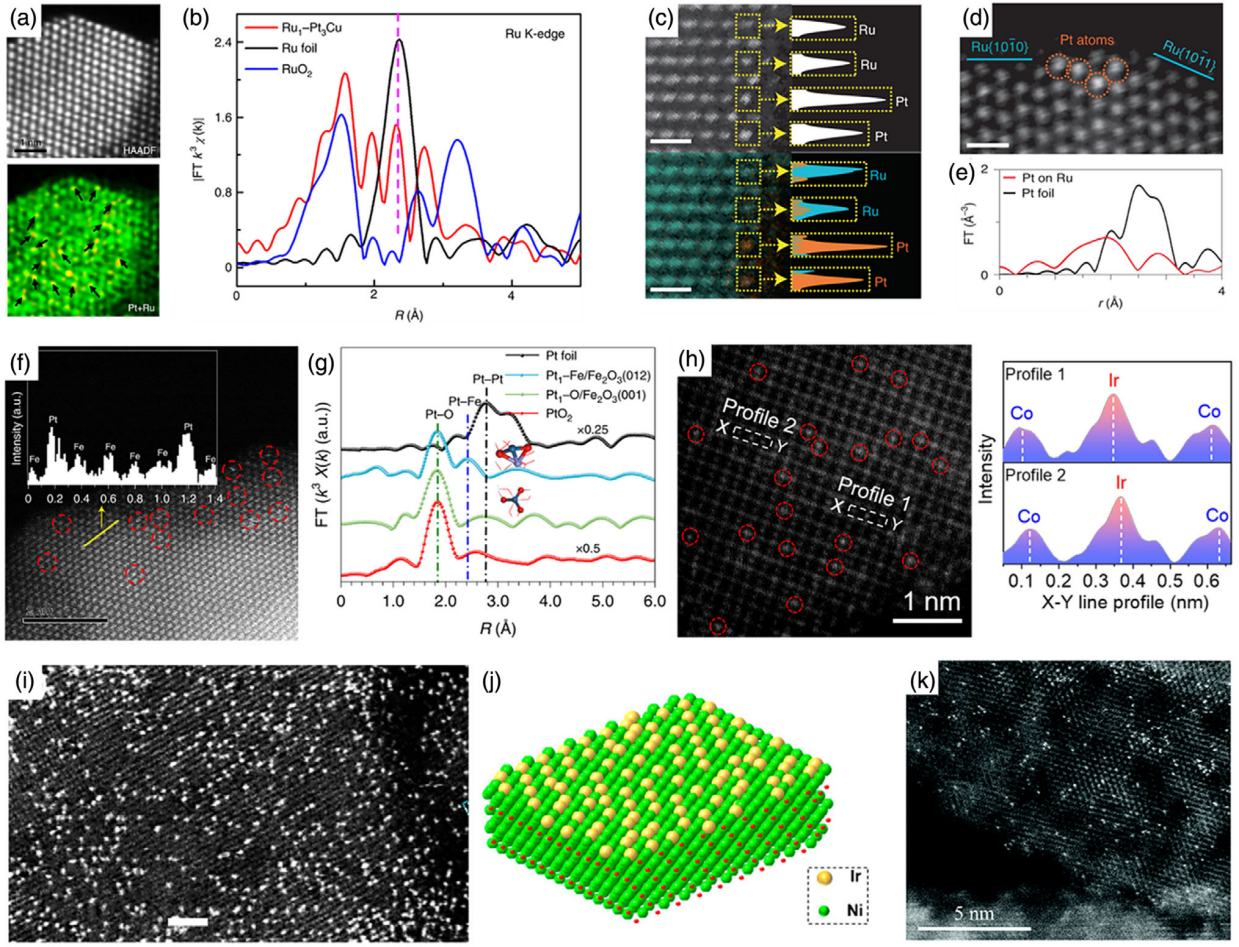
a) HAADF‐STEM image and elemental mapping of the Ru_1_–Pt_3_Cu. b) Ru K‐edge EXAFS spectra of the Ru_1_–Pt_3_Cu catalyst. Reproduced with permission.^[^ ^85^ ^]^ Copyright 2019, Springer Nature. c) HAADF‐STEM image (top) and EDX mappings (bottom) and corresponding plots of the intensity of the bright contrast spots (white), the single Pt atoms (orange), and Ru atoms (blue) identified in the yellow boxes. d) HAADF‐STEM image shows the position of Pt atoms in relation to Ru facets. Scale bars: 0.5 nm. e) EXAFS spectra of Pt foil and single Pt atoms on Ru nanoparticles. c–e) Reproduced with permission.^[^ ^79^ ^]^ Copyright 2022, The Authors, published by Springer Nature. f) An AC HAADF‐STEM image of Pt_1_–Fe/Fe_2_O_3_ (012), with single‐site Pt marked by the red dashed circles. The inset shows intensity profiles along the yellow line. g) Pt L_3_‐edge EXAFS spectra of the Pt_1_–Fe/Fe_2_O_3_ (012). f,g) Reproduced with permission.^[^ ^98^ ^]^ Copyright 2021, The Authors, published by Springer Nature. h) Magnified AC HAADF‐HRTEM image of Ir–Co_3_O_4_ with red circles showing isolated Ir single atoms stabilized on Co_3_O_4_ and corresponding atomic intensity profile obtained at profiles 1 and 2. Reproduced under the terms of the CC‐BY Creative Commons Attribution 4.0 International license (https://creativecommons.org/licenses/by/4.0).^[^ ^99^ ^]^ Copyright 2022, The Authors, published by Springer Nature. i) HAADF‐STEM images of the Ir–NiO catalyst, in which the bright spots are ascribed to Ir single atoms. j) Corresponding atomic models. i,j) Reproduced with permission.^[^ ^100^ ^]^ Copyright 2020, American Chemical Society. k) HAADF‐STEM image of Ni_3_Fe–CO_3_^2−^ LDH–Pt SA nanosheet. Reproduced with permission.^[^ ^101^ ^]^ Copyright 2021, Royal Society of Chemistry.

Li et al. successfully employed a Pt_3_Cu alloy loaded with single Ru atoms (Ru_1_–Pt_3_Cu) to catalyze the OER.^[^ ^85^ ^]^ The results obtained using high‐angle annular dark‐field scanning transmission electron microscopy (HAADF‐STEM) illustrated that the Ru atoms were uniformly dispersed and well separated on the Pt_3_Cu matrix, as indicated by the arrows in **Figure** **1**a. Extended Ru K‐edge X‐ray absorption fine structure (EXAFS) analysis was performed, and the results verified the isolation of Ru atoms from the Pt_3_Cu supports and the existence of efficient metal–metal bond interactions (Figure 1b). Ru_1_–Pt_3_Cu displays high activity and strong resistance to dissolution in the acidic OER, mainly due to the precise optimization of the electronic structures of the Ru single atoms by the Pt_3_Cu alloy to lower the overpotential, and the Pt–Ru bonds restrict the leaching of active Ru species. In addition, Poerwoprajitno et al. designed a promising catalyst by supporting single Pt atoms on Ru particles.^[^ ^79^ ^]^ Atomic‐resolution HAADF‐STEM coupled with energy‐dispersive X‐ray (EDX) mapping showed that isolated Pt atoms were located at the support edge and remained in the same columns as Ru (Figure 1c,d). The presence of the Pt–Ru bond in the EXAFS confirmed the close interaction between the single atoms and the supports (Figure 1e). Pure Pt usually suffers from CO poisoning, which results in poor performance for the methanol oxidation reaction (MOR). In this study, the generated OH^−^ on the Ru support effectively removed the CO adsorbed on the Pt atoms, and the strong Pt–Ru bonding interactions resulted in high activity and stability. Chang et al. used a catalyst to accelerate the ethanol oxidation reaction (EOR) effectively by downsizing rhodium (Rh) into single atoms and loading them onto Pt nanocubes (Rh_at_O–Pt NCs).^[^ ^86^ ^]^ Both HAADF‐STEM and Rh K‐edge EXAFS indicated that the isolated Rh atoms were situated on the surface of the Pt NCs. Consequently, the alleviated poisoning effect on Rh_at_O–Pt NCs was accomplished by the Rh single atoms, and the synergistic function of Rh and Pt promoted the complete oxidation of ethanol, collectively boosting the EOR. Wang et al. supported monodispersed gallium (Ga) atoms on Pt_3_Mn nanocrystals (Ga–O–Pt_3_Mn) to efficiently drive the EOR.^[^ ^87^ ^]^ An investigation of the mechanism revealed that unconventional p–d orbital hybridization is induced between Ga single atoms and Pt_3_Mn supports, which is beneficial for EOR enhancement. Besides these studies, several other novel SAAs catalysts supporting metals or alloys with excellent catalytic performances, such as Fe–Pb, Ni–Ru, Pt–Ni_3_Fe, and In–Pt, have also been reported.^[^ ^88^, ^89^, ^90^, ^91^ ^]^ Despite the considerable progress recently made in this field, SAAs are still a very new type of SACs, with more effort required to explore their properties in varying metal combinations and toward diverse green energy conversion.

### 2.2 Metal Oxides/Metal Hydroxides

Metal oxides and metal hydroxides, including spinels, perovskites, and layered double hydroxides (LDH), are indispensable candidates for various catalytic reactions because of their distinct properties, such as surface acidity/basicity and redox features, as well as their flexible structures.^[^ ^92^, ^93^, ^94^ ^]^ These materials can offer abundant anchoring sites for supporting single atoms, owing to the defects (steps, vacancies) being easily constructed on metal oxide surfaces and the –OH groups getting enriched on metal hydroxide surfaces. Meanwhile, metal oxides and hydroxides usually have larger specific areas than other supports, which provide more possibilities for constructing high‐loading SACs.^[^ ^95^, ^96^, ^97^ ^]^ So far, many types of metal oxides/metal hydroxides with an ample choice of electron configurations, crystal structures, and defects have been explored as supports for SACs and are widely applied in electrochemical redox reactions.

For example, Gao et al. used monodisperse Pt atoms on Fe_2_O_3_ (012) (Pt_1_–Fe/Fe_2_O_3_ (012)) to construct highly active and stable ORR catalysts.^[^ ^98^ ^]^ As shown in Figure 1f, the HAADF‐STEM image displays individual Pt atoms on Fe_2_O_3_, further confirmed by the atomic intensity profile. The EXAFS results (Figure 1g) further reveal that no Pt–Pt bonds are present, whereas the appearance of the obvious Pt–Fe peak indicates a strong metal–support interaction. Pt_1_–Fe/Fe_2_O_3_ (012) exhibits excellent activity in both ORR and practical fuel cells due to its strong electronic coupling. In addition, our group achieved Ir single atoms (1.05 at%) on a Co_3_O_4_ support (Ir–Co_3_O_4_).^[^ ^99^ ^]^ The magnified AC HAADF‐STEM image clearly demonstrates that high‐density bright Ir spots were uniformly dispersed on Co_3_O_4_, further corroborated by the atomic intensity profiles obtained at sites a and b (Figure 1h). As revealed by both operando X‐ray absorption spectroscopy (XAS) investigations and calculations, Ir and Co atoms, with their bridged electrophilic O ligands, act synergistically as active sites in Ir–Co_3_O_4_, jointly contributing to the excellent OER activity and stability toward acidic OER. In addition, Gu et al. reported an unprecedented high loading of Ir single atoms (18 wt%) on a NiO support via the formation of Ir–O covalent bonding interactions.^[^ ^100^ ^]^ The AC‐STEM image and simulated model show that dense Ir atoms are likely located on the outermost NiO surface (Figure 1i,j). The reactive Ir single atoms with high loading can activate NiO, leading to a drastically improved performance in the OER. Furthermore, LDH with the 2D structure and confined space is an ideal support for dispersing SACs. Wang et al. proposed a general strategy for intercalating single Pt atoms into Ni_3_Fe LDH supports (Ni_3_Fe–CO_3_^2−^ LDH–Pt SA), as shown in Figure 1k.^[^ ^101^ ^]^ The catalytic results show that Pt single atoms and Ni_3_Fe LDH are active toward HER and OER, respectively. As a result, an alternating synergy function is introduced in the Ni_3_Fe–CO_3_^2−^ LDH–Pt SA, which improves the improved overall water splitting activity. Ni(OH)_2_‐supported Ir single atoms catalyst (Ir_1_–Ni(OH)_2_) with a remarkable OER stability was also reported by Song's group.^[^ ^102^ ^]^

### 2.3 Metal‐Derived Compounds

Generally, the coordination of donors from different supports can influence the coordination environment and electronic properties of isolated metal atoms, which can impact catalytic performance by regulating interactions with intermediates.^[^ ^103^ ^]^ Inspired by this knowledge, some researchers have gradually focused on metal‐derived compounds, including chalcogenides, phosphides, selenides, and nitrides, which can serve as supports for providing the coordination elements sulfur (S), phosphorus (P), selenium (Se), and nitrogen (N).^[^ ^104^ ^]^ These coordinated heteroatoms possess multiple valence states and can precisely adjust the coordination number and affect the charge density of single atoms by electron transfer. Additionally, these metal‐derived compounds exhibit high conductivity and strong corrosion resistance in electrochemical environments, leading to good activity and stability.^[^ ^105^ ^]^ Due to these advantages, recent research has focused on metal‐derived compounds as the desired supports for single metal atoms.

**Figure 2:**
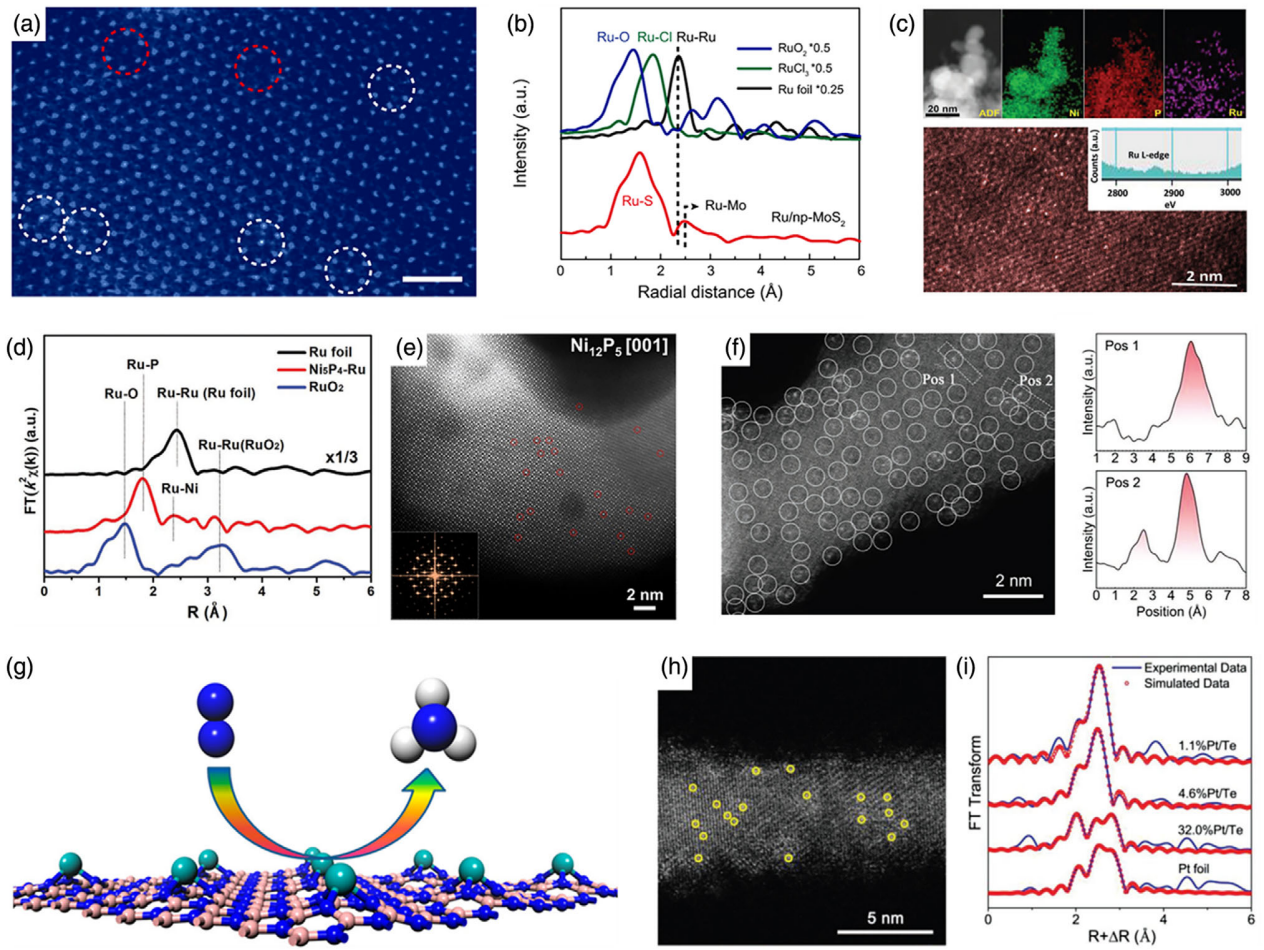
a) HAADF‐STEM image and b) Ru K‐edge EXAFS spectra of Ru/np‐MoS_2_. a,b) Reproduced under the terms of the CC‐BY Creative Commons Attribution 4.0 International license (https://creativecommons.org/licenses/by/4.0).^[^ ^106^ ^]^ Copyright 2021, The Authors, published by Springer Nature. c) Elemental mappings and HAADF‐STEM image of Ni_5_P_4_–Ru (inset: EELS spectrum at Ru L‐edge). d) The Ru K‐edge EXAFS of Ni_5_P_4_–Ru. c,d) Reproduced with permission.^[^ ^107^ ^]^ Copyright 2020, Wiley‐VCH. e) HAADF‐STEM image and FFT (inset) of Ir_SA_–Ni_2_P. Reproduced with permission.^[^ ^108^ ^]^ Copyright 2021, American Chemical Society. f) AC HAADF‐STEM image of Pt_1_–VN (Pt single atoms are marked by white circles), and the line profiles of intensity obtained in Pos 1 and Pos 2. Reproduced with permission.^[^ ^110^ ^]^ Copyright 2022, Wiley‐VCH. g) Atomic model of single Mo atom supported on defective BN. Reproduced with permission.^[^ ^117^ ^]^ Copyright 2017, American Chemical Society. h) HAADF‐STEM image and i) corresponding Pt L_3_‐edge EXAFS spectra of 1.1%Pt/Te. h,j) Reproduced with permission.^[^ ^118^ ^]^ Copyright 2019, Wiley‐VCH.

MoS_2_ is a cost‐effective metal chalcogenide that has been extensively studied for the HER; however, its activity is still unsatisfactory. Tan et al. reported an electrocatalyst that supports single Ru atoms on MoS_2_ with sulfur vacancies (SVs) (Ru/np‐MoS_2_) to enhance the intrinsic HER activity.^[^ ^106^ ^]^ In **Figure** **2**a, the HAADF‐STEM image clearly displays homogeneously dispersed Ru atoms and defects on the MoS_2_ support. The corresponding Ru K‐edge EXAFS spectrum shows prominent peaks assigned to Ru–S and Ru–Mo scattering, indicating that single Ru atoms were coordinated by S (Figure 2b). Consequently, the synergistic function introduced in Ru/np‐MoS_2_ led to a remarkable alkaline HER performance. Bifunctional metal phosphides have already been proven to activate both the HER and OER, and can serve as platforms for trapping single atoms. Song et al. synthesized a Ni_5_P_4_ catalyst incorporating single Ru atoms (Ni_5_P_4_–Ru) by filling the surface defects on the supports with Ru atoms.^[^ ^107^ ^]^ As shown in Figure 2c, the presence of single Ru atoms was confirmed by HAADF‐STEM. The presence of Ru–P and Ru–Ni bonds in Figure 2d indicates the atomically dispersed Ru being stabilized by the surrounding P atoms and that the optimized Ru electronic structure plays a vital role in the competitive HER activity. Besides the HER, Gu et al. also reported an Ir single‐atom‐doped Ni_2_P catalyst (Ir_SA_–Ni_2_P) for efficient OER.^[^ ^108^ ^]^ Owing to the reconstructed Ir–O–P bonding environments, Ir_SA_–Ni_2_P possesses an optimal intermediate binding energy, contributing to a marked improvement in the OER activity (Figure 2e). Moreover, metal selenides with high electrical conductivity are favorable for electrocatalytic reactions. Therefore, the Pt single atoms doped nanoporous Co_0.85_Se (Pt/np‐Co_0.85_Se) was explored by Tan and co‐workers as an outperformed catalyst for HER.^[^ ^109^ ^]^ Studies have indicated that the enhanced HER is mainly because of the activated surface states of Co_0.85_Se and the reduced energy barrier caused by the coordinated Se atoms. In addition, metal nitrides with good hydrophilicity and corrosion resistance are promising candidates for long‐term electrocatalytic operation. Inspired by this finding, Xie et al. successfully explored pore‐rich VN supports with abundant unsaturated N atoms to stabilize single Pt atoms.^[^ ^110^ ^]^ From the related HAADF‐STEM image and line profiles, it is clear that it is atomically distributed on the VN support (Figure 2f). The HER experimental results confirmed that both enhanced activity and prolonged stability were achieved with the prepared catalyst. Other metal‐derived compound‐supported SACs, such as Pt–MoC and Ru–NiCo_2_S_4_, have also been developed for energy‐related applications.^[^ ^111^, ^112^ ^]^

### 2.4 Nonmetal Compounds

Nonmetal compounds with high thermal conductivity, strong abrasive resistance, and low dielectric constants are commercially applied in the steel processing industry. For example, scientists have recently found that the outstanding physical and chemical durability of boron nitride (BN) makes it suitable for supporting single metal atoms, which can greatly improve the electrical conductivity and catalytic performance of SACs.^[^ ^113^, ^114^, ^115^ ^]^ Jiang et al. developed a seeding strategy to prepare single nickel (Ni) atoms on BN for the electrochemical conversion of carbon dioxide (CO_2_) to CO.^[^ ^116^ ^]^ Furthermore, Chen et al. explored the possibility of dispersing single Mo atoms on BN as efficient electrocatalysts for the NRR through theoretical calculations (Figure 2g).^[^ ^117^ ^]^ In addition, other nonmetal compounds with unique properties have been explored as supports for loading SACs to accelerate diverse energy conversion applications. As depicted in Figure 2h,i, pure tellurium (Te) nanowires are used as supports in the study of the as‐obtained 1.1% Pt/Te. Pt single atoms were observed from the brighter spots in HAADF‐STEM, affirmed by the EXAFS results.^[^ ^118^ ^]^ Considering the strong mutual interaction between Pt and Te, 1.1% Pt/Te exhibited an enhanced performance in formic acid dehydrogenation at room temperature. Silicon dioxide (SiO_2_) is regarded as a suitable carrier for single metal atoms due to its ordered pore structure and precise designability. Wu et al. prepared a catalyst by trapping single Co atoms in ordered SiO_2_ channels to boost nonoxidative propane dehydrogenation.^[^ ^119^ ^]^ Subsequently, Pennycook et al. successfully synthesized single Hf atoms within a SiO_2_ interlayer.^[^ ^120^ ^]^

The main concern regarding non‐C supports is their intrinsic electrical conductivity, which is closely related to their electrocatalytic performance. Pure metals and alloys possess excellent conductivity, making the formed SAAs beneficial for electrocatalytic reactions and exhibiting extremely low resistance.^[^ ^79^, ^85^ ^]^ Metal oxides, conversely, are generally ionic crystals and show poor conductivity. However, constructing defects and anchoring single metal atoms on oxides can effectively accelerate the electronic flow of the overall materials, thereby enhancing the metal–support interactions in catalysis.^[^ ^99^ ^]^ In contrast, many metal‐derived compounds, including sulfides and phosphides, show better conductivity than oxides because of the lower electronegativity and bonding energy of P and S. Because of this, single atoms incorporating metal sulfides (such as MoS_2_) and metal phosphides (such as Ni_5_P_4_) being promising candidates for electrocatalytic HER and OER.^[^ ^106^, ^107^ ^]^ Most nonmetal compounds exhibit poor electrical conductivity due to the lack of conductive chemical bonds. It is difficult to optimize the overall electronic transport rate by loading single metal atoms on these supports. Thus, they are usually employed in electrocatalytic reactions such as the ECR and NRR, which focus on selectivity rather than activity.^[^ ^116^ ^]^ One crucial point to note is that, regardless of the electrical conductivity of the non‐C supports, the catalysts should be loaded on the treated C before the electrocatalytic reactions. This further increases the electron transport rate and significantly prevents the agglomeration of electrocatalysts during the catalytic process.

## 3 Synthesized Methods for Non‐C‐Supported SACs

Researchers have explored many approaches for synthesizing SACs over decades of development, summarized into two categories: bottom‐up and top‐down methods.^[^ ^121^ ^]^ The bottom‐up method involves the adsorption of precursors on solid supports, such as immersion, followed by a reduction to SACs. This method includes hydrothermal reactions, impregnation, and electrochemical and photochemical deposition. The top‐down method usually refers to using nanoparticles supported on substrates as target precursors and their transformation into single atoms under specific conditions, such as high‐temperature pyrolysis or a strong reducing atmosphere.^[^ ^122^ ^]^ Both approaches have advantages and disadvantages. The facile bottom‐up method is extensively employed in most studies; however, the excessive addition of precursors increases the cost, and it is difficult to regulate the microenvironment of single metal atoms. The synthesis requirements for the top‐down method are harsh, but the structure of SACs can be regulated precisely. In this section, we summarize the current preparation methods for non‐C‐supported SACs and analyze their advantages and disadvantages.

### 3.1 Hydrothermal Method

**Figure 3:**
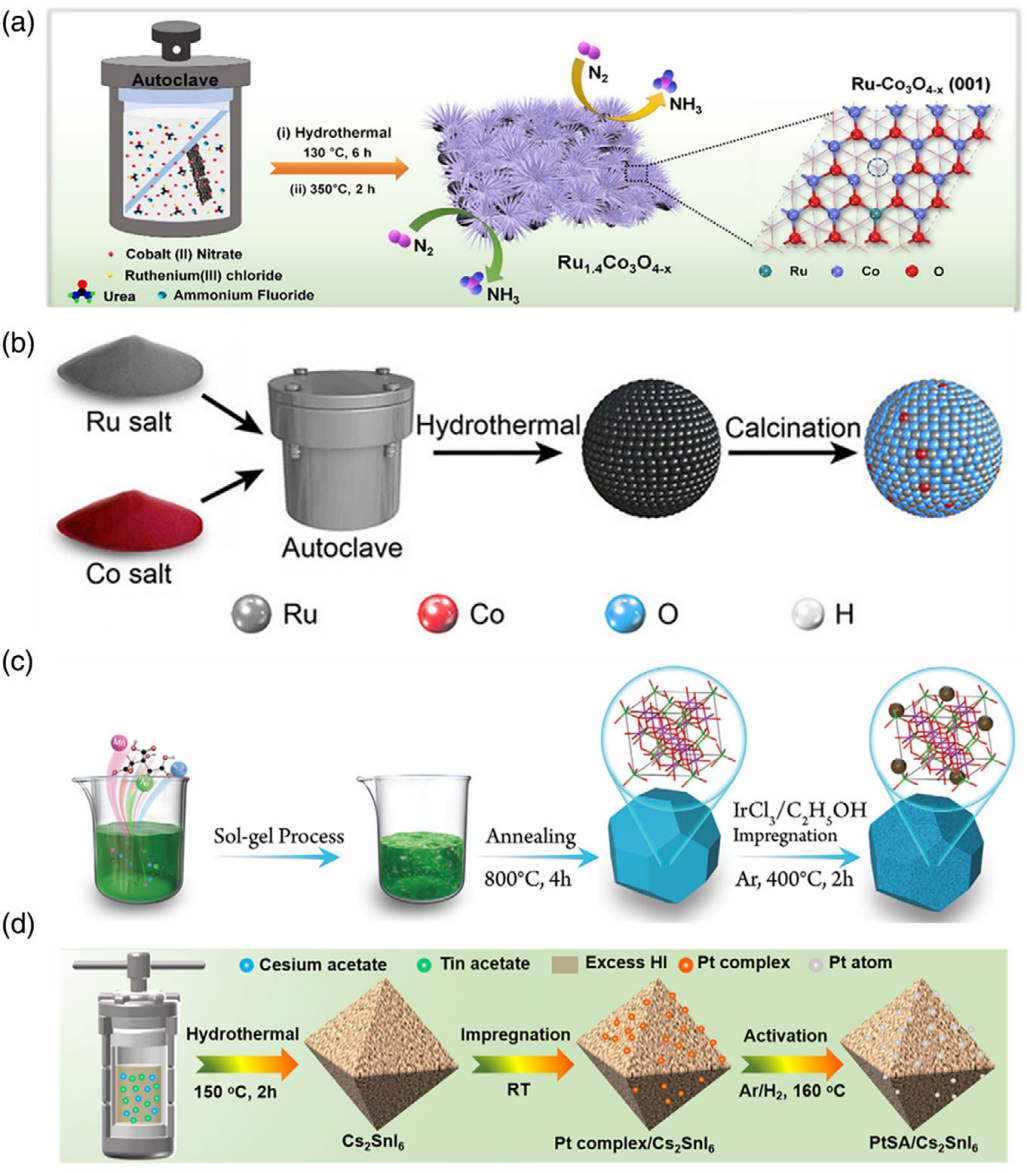
a) Schematic illustration of the fabrication of Ru–Co_3_O_4–*x*_ nanowires via hydrothermal reaction. Reproduced with permission.^[^ ^126^ ^]^ Copyright 2021, American Chemical Society. b) Schematic illustration of the synthesis of Co single‐atom‐incorporated RuO_2_ sphere. Reproduced with permission.^[^ ^127^ ^]^ Copyright 2021, Wiley‐VCH. c) The schematic diagram for the synthetic procedure of the Ir_1_/Ni_1.6_Mn_1.4_O_4_. Reproduced with permission.^[^ ^131^ ^]^ Copyright 2022, Wiley‐VCH. d) Schematic diagram of the preparation process of the PtSA/Cs_2_SnI_6_ catalyst. Reproduced under the terms of the CC‐BY Creative Commons Attribution 4.0 International license (https://creativecommons.org/licenses/by/4.0).^[^ ^132^ ^]^ Copyright 2021, The Authors, published by Springer Nature.

The hydrothermal method involves dissolving and recrystallizing precursors in a sealed vessel using organic matter as the solvent, where the driving force is high pressure or the addition of a reducing agent. Recently, researchers found that the hydrothermal method is reliable for preparing SACs because it does not require extremely high temperatures and avoids agglomeration. Additionally, the morphology of the synthesized nanomaterials can be controlled by regulating different types of reducing agents, solvents, and metal precursors. Moreover, single metal atoms and supports can be simultaneously prepared in one pot to prevent the waste caused by multiple synthetic steps.^[^ ^123^, ^124^, ^125^ ^]^ Novel non‐C‐supported SACs prepared by the hydrothermal method have been explored for various energy‐related applications because of these benefits. For example, Lee and co‐workers synthesized the Co‐based nanowires doped with varying amounts of Ru by placing a piece of C cloth in an autoclave containing Co(NO_3_)_2_⋅6H_2_O and Ru(Cl)_3_⋅nH_2_O in 40 mL of DI water (**Figure** **3**a). Then, urea and NH_4_F were added as reducing agents, with the mixture heated to 130 °C for 6 h using a muffle furnace for hydrothermal reaction.^[^ ^126^ ^]^ Eventually, Ru single‐atom‐doped Co_3_O_4−*x*_ (Ru_*y*_Co_3_O_4−*x*_) was obtained by further calcination in air and exhibited excellent NRR activity with a high NH_3_ Faradaic efficiency (FE) and good stability, outperforming commercial Ru/C. Li et al. successfully introduced single Co atoms into RuO_2_ (Co‐SAC/RuO_2_) via a hydrothermal process (Figure 3b).^[^ ^127^ ^]^ Specifically, RuCl_3_ and CoCl_3_ were homogeneously dissolved in water, and the mixture was stirred, followed by adding urea and SDBS. Then, the mixture was transferred into an autoclave and kept at 100 °C for 6 h. Subsequently, the sample was collected and calcined in air to get Co‐SAC/RuO_2_. This catalyst has promising performance for both the HER and OER, which can be attributed to the tailored electronic structure of RuO_2_ caused by the doped Co single atoms. Besides, Wang et al. prepared the Pt_3_Mn nanomaterials and dispersed them in the mixture of water and ethanol, followed by mixing GaCl_3_ with the above solution and heating it up to 200 °C for 2 h.^[^ ^87^ ^]^ In this process, ethanol played a vital role in reducing the Ga precursors to single atoms. As a result, they constructed monodisperse Ga atoms on Pt_3_Mn nanocrystals, which showed high EOR activity. Many other non‐C‐supported SACs, such as Co–V_2_O_5_, have been explored using similar methods.^[^ ^128^ ^]^ Although many research milestones have been achieved using the hydrothermal method, a large amount of metal precursors may be wasted in the synthesis process, leading to a low loading of single atoms. To address this problem, it may be necessary to add additional complexing agents to the solvent or modify more anchoring sites on the support in advance. The hydrothermal method, with an additional solvent and reducing agent, may also affect the catalytic performance and result in a higher cost than other methods. In addition, the single metal atoms generated by the hydrothermal method are present not only on the surface but also inside the supports, making it difficult to achieve full atomic utilization.

### 3.2 Impregnation Method

As one of the most classical synthesis methods for SACs, the impregnation method involves immersing the support materials into an aqueous solution of metal salts, removing the excess solution, and obtaining the catalyst by sequential drying, calcining, and activation. This method strongly depends on the adsorption capacity of the metal salts on the support surface; hence, the loading amount and dispersion degree are closely related to the metal–support interactions and support characteristics.^[^ ^129^ ^]^ Compared with the hydrothermal method, the single metal atoms prepared by impregnation can achieve maximum atomic utilization in reactions, as the single metal atoms prepared by impregnation cover the surface of the support. The most significant advantage of this facile approach is that it can be used to synthesize almost all types of SACs.^[^ ^130^ ^]^ Chen and co‐workers synthesized Ni_1.6_Mn_1.4_O_4_ immobilized with Ir single atoms (Ir_1_/Ni_1.6_Mn_1.4_O_4_) via the impregnation method.^[^ ^131^ ^]^ As shown in Figure 3c, the support of spinel Ni_1.6_Mn_1.4_O_4_ was first synthesized using the sol–gel method and dispersed uniformly in ethanol. Subsequently, IrCl_3_ solution was poured into the above dispersion dropwise, and Ir_1_/Ni_1.6_Mn_1.4_O_4_ was obtained by thermal treating at 400 °C for 2 h under argon (Ar). Ir_1_/Ni_1.6_Mn_1.4_O_4_ exhibited enhanced performance in alkaline seawater splitting, benefiting from the fully exposed single Ir atoms. Additionally, a Pt single‐atom‐doped Cs_2_SnI_6_ (Pt SA/Cs_2_SnI_6_) catalyst was discovered by Guo et al. via the impregnation method, and the synthetic procedure is shown in Figure 3d.^[^ ^132^ ^]^ In brief, Cs_2_SnI_6_ was prepared and impregnated with a Pt complex. Subsequently, Pt SA/Cs_2_SnI_6_ was successfully produced by calcination. Recently, many novel non‐C‐supported SACs, such as Ir–Ni(OH)_2_, Pt–Mo_2_C, Mo–Pt/NC, and Ru–CoFe LDH, have been successfully explored using the impregnation method to investigate the relationship between high activity and surface chemistry in energy conversion.^[^ ^102^, ^111^, ^133^, ^134^ ^]^ Nevertheless, physical and chemical adsorption are the major intrinsic driving forces for the impregnation method to capture metal atoms into the anchoring sites of the support; hence, the obtained non‐C‐supported SACs might display poor thermal and chemical durability. Additionally, calcination and decomposition processes require a large amount of energy and produce exhaust gas pollution, which restricts their large‐scale application. Moreover, the biggest challenge is increasing the loading amount and accelerating the loading rate of single metal atoms on supports using this method.

### 3.3 Electrochemical/Photochemical Deposition Method

**Figure 4:**
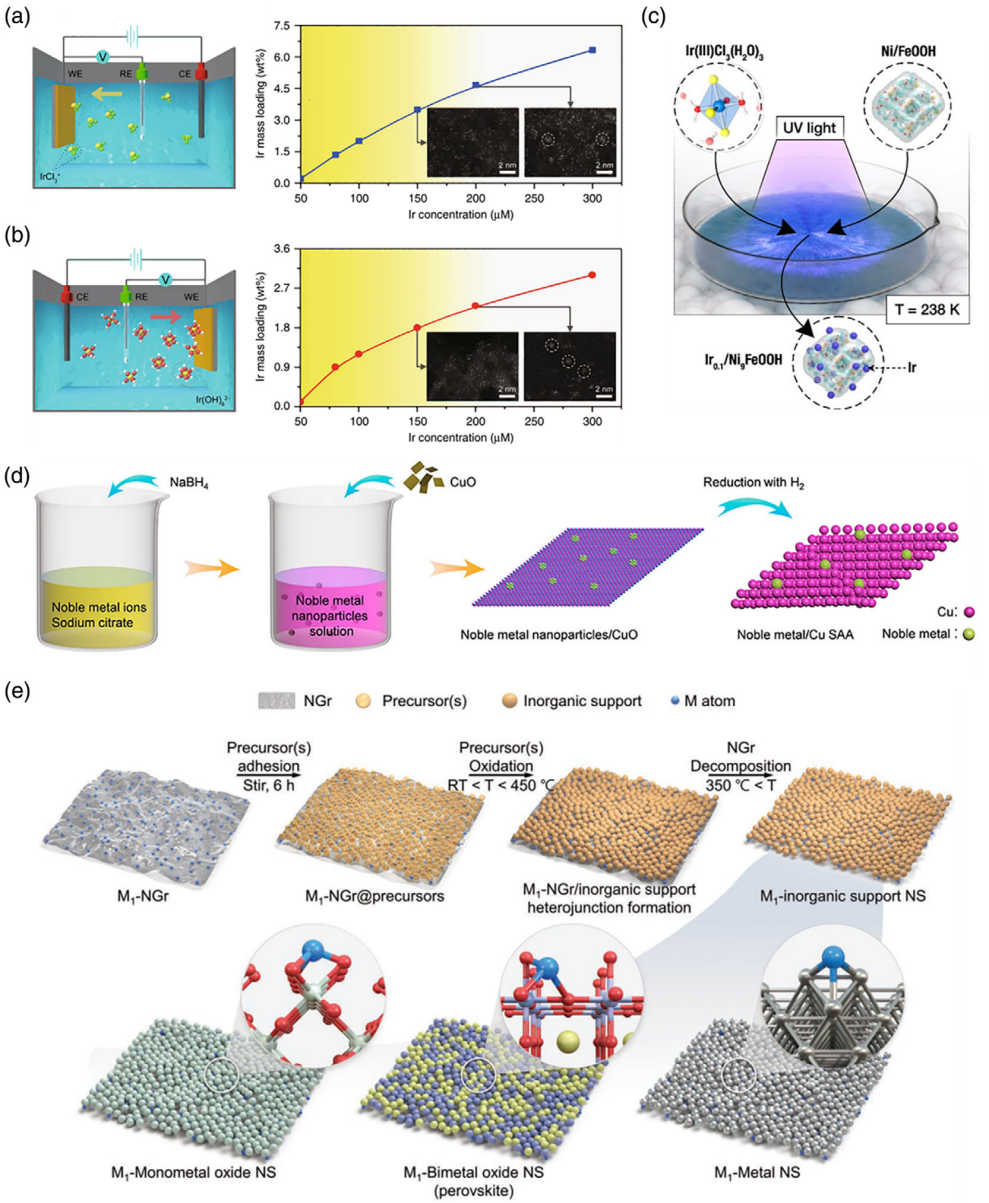
a,b) Schematic of cathodic (a) and anodic (b) deposition of Ir species. The yellow, green, red, and white spheres represent Ir, Cl, O, and H atoms, respectively, and the corresponding Ir mass loadings as a function of Ir concentration in the 1 M KOH for cathodic and anodic deposition. The inset images correspond to the HAADF‐STEM images obtained at a certain concentration. a,b) Reproduced under the terms of the CC‐BY Creative Commons Attribution 4.0 International license (https://creativecommons.org/licenses/by/4.0).^[^ ^138^ ^]^ Copyright 2020, The Authors, published by Springer Nature. c) In situ cryogenic‐photochemical reduction synthesis of Ir_0.1_/Ni_9_FeOOH samples. Reproduced with permission.^[^ ^139^ ^]^ Copyright 2021, National Academy of Sciences. d) Schematic illustration of the transformation of noble metal nanoparticles to SAAs. Reproduced with permission.^[^ ^140^ ^]^ Copyright 2022, American Chemical Society. e) Schematic illustration for the synthesis of Pt single atoms stabilized on various inorganic (metal oxide, perovskite, and metallic) nanosheets via the N‐doped graphene sacrificial template route. Reproduced with permission.^[^ ^141^ ^]^ Copyright 2022, Wiley‐VCH.

The versatile electrochemical deposition method is defined as the reduction of the metal precursors via electricity and their subsequent deposition onto support. This method has recently gained increasing attention for the construction of a variety of SACs with differing compositions and properties. The electrochemical deposition method is environmentally friendly and nonenergy‐intensive compared to the thermochemical method. It can be performed in a simple three‐electrode cell with a mild current input at room temperature. The fast kinetics of electrodeposition can significantly improve the efficiency of the deposition process when compared to other time‐consuming methods. Additionally, the parameters of SACs synthesized by electrochemical deposition can be precisely regulated on electrochemical workstations, allowing for easy control of the dispersion degree and loading mass of SACs.^[^ ^135^, ^136^ ^]^ Generally, potential cycling, linear polarization scanning, and constant potential electrolysis are the three different methods used for the practical operation of electrochemical deposition. For instance, Cao et al. utilized the cyclic voltammetry (CV) method to fabricate single Ir atoms on a NiFeS nanosheet array substrate (Ir_1_/NFS).^[^ ^137^ ^]^ NFS was first generated on a Ni foam electrode by sweeping the potential in a solution containing thiourea and Ni–Fe precursors. Then, the Ir precursor was dropped into the electrolyte, and the electrode was swept between 0.3 and −0.3 V versus Hg/HgO for depositing Ir single atoms. Notably, Ir atoms were deposited on the NFS substrate surface, maximizing their utilization and delivering an ultralow overpotential for the OER. An interesting phenomenon in the electrodeposition approach is that the electronic properties of the same single atom are different via cathodic or anodic deposition, providing SACs with different catalytic performances. Zeng et al. proposed a representative illustration of the cathodic and anodic depositions of Ir single atoms on Co(OH)_2_ in a KOH electrolyte (denoted as C‐Ir_1_/Co(OH)_2_ and A‐Ir_1_/Co(OH)_2_, respectively) as shown in **Figure** **4**a,b.^[^ ^138^ ^]^ The corresponding syntheses were conducted from 0.10 to −0.40 V for cathodic deposition and from 1.10 to 1.80 V for anodic deposition to obtain the SACs, respectively. They also found that when the critical concentration was reached, single Ir atoms gathered as clusters. Catalytic experiments showed that C‐Ir_1_/Co(OH)_2_ could efficiently catalyze the HER, while A‐Ir_1_/Co(OH)_2_ was a promising candidate for the OER. Although electrochemical deposition is suitable for synthesizing non‐C‐supported SACs, some supports are not suitable for this approach due to reconstruction or leaching at high voltages in corrosive electrolytes. The photochemical deposition procedure is similar to electrochemical deposition, except that UV light is commonly used as an energy source to drive the formation of single atoms on the support. As depicted in Figure 4c, the Ir single atom doped Ni_9_FeOOH (Ir_0.1_/Ni_9_FeOOH) was successfully synthesized by mixing the IrCl_3_·3H_2_O and NiFe oxyhydroxides solution followed with UV irradiation at −35 °C for 1 h.^[^ ^139^ ^]^ The obtained Ir_0.1_/Ni_9_FeOOH exhibits high OER activity and outperforms commercial IrO_2_. In summary, the electrochemical/photochemical deposition method is a recently emerging, facile synthesis technique requiring much research effort to fabricate more efficient non‐C‐supported SACs.

### 3.4 Other Methods

In addition to the abovementioned strategies, several other innovative synthetic strategies (H_2_ reduction, sacrificial templates, and mechanochemical methods) have been developed for fabricating single metal atoms on non‐C supports.^[^ ^121^ ^]^ This section provides a brief overview of the new protocols. H_2_ reduction methods are gradually gaining research attention because of the use of reducing gases and high temperatures to accelerate atomic migration from nanoparticles to isolated metal atoms on supports. Yu et al. achieved novel catalysts by the thermal treatment of noble metal nanoparticles on CuO supports with the assistance of H_2_/Ar (Figure 4d).^[^ ^140^ ^]^ In this process, CuO supports were well dispersed in a solution containing noble metal nanoparticles. Then, the mixture was dried and heated in a furnace with H_2_ flowing for 1 h at 200 °C to obtain the noble metal single atoms doped Cu nanomaterials. The corresponding mechanism is that dissolved H_2_ can cause lattice expansion of the bulk noble metal, weakening the metal–metal bonds and leading to the formation of isolated atoms. It is worth noting that this approach is limited in some non‐C supports, which may result in the reduction of oxides to metals/alloys under strong reducing atmospheres. Additionally, there is a high demand for a reliable, general synthetic approach for non‐C‐supported SACs that can stabilize high loadings of single atoms. Kim et al. demonstrated a general sacrificial template method using single‐atom‐stabilized graphene as a template for synthesizing high‐loading SACs on various supports, including metal (Pd), metal oxide (SnO_2_, ZnO, and Co_3_O_4_), and perovskites (LaCoO_3_) (Figure 4e).^[^ ^141^ ^]^ Initially, the single‐atom‐stabilized N‐doped graphene (M_1_–NGr) was prepared by pyrolyzing EDTA‐2Na⋅2H_2_O. Then, M precursors (M = Sn, Zn, La, Co, Pd) were simultaneously anchored on M_1_–NGr, and the mixture was calcined in air to thermally decompose the NGr and oxidize the M precursors. Finally, M_1_ single‐atom‐loaded M oxide supports were obtained. This novel approach is highly competitive for producing high‐loading non‐C‐supported SACs with excellent thermal stability and general applicability. Furthermore, mechanochemical methods such as ball milling offer a new way to realize the mass production of SACs driven mainly by mechanical energy. Recently, Ji et al. and Ma et al. reported Pt single‐atom‐alloyed Co and Pd single‐atom‐supported ZnO catalysts, respectively, using the ball‐milling method, which can achieve mass production at the kilogram scale.^[^ ^142^, ^143^ ^]^ Notably, the main advantage of the mechanochemical method is that almost no additives are needed, which simplifies preparation steps and reduces costs, making it promising for practical applications in industry.

## 4 Metal–Support Interaction

Strong metal–support interactions were first discovered by Tauster et al. proposing that the chemical bonding between noble metals and TiO_2_ increases catalytic activity.^[^ ^144^ ^]^ Since then, these interactions between metals and supports have been the focus of attention for high‐performance heterogeneous catalysts. SACs are the most representative supported nanocatalysts, and their construction and catalytic properties depend on the metal–support interactions. Non‐C‐supported SACs have an advantage over C‐supported SACs because they derive from diverse and feasible supports, resulting in more abundant and efficient metal–support interactions.^[^ ^67^, ^68^, ^69^ ^]^ Recent research has identified three typical interactions that can be principally generalized to electron density redistribution, covalent bonding, and synergistic catalysis.

### 4.1 Electron Density Redistribution

In 2012, Campbell utilized density functional theory (DFT) calculations to determine the interaction between Pt and a cerium‐based support, based on which they found electron density redistribution at the interface and named it the electronic metal–support interaction.^[^ ^145^ ^]^ Generally, electron density redistribution is associated with a strong capability to rehybridize the orbitals, modulate electronic structures, particularly the *d*‐band center of the active sites, and decrease reaction barriers, thus optimizing intermediate binding energy. SACs that have close interaction between single atoms and supports, as well as the diverse electronic environments in non‐C‐supported SACs, make them ideal systems for investigating the influence of electron density redistribution. And the direction of electron transfer is closely related to the coordination environment of single atoms. Chen et al. discovered the electronic structures of isolated Pt atoms on MoSe_2_ (Pt–SAs/MoSe_2_) using XAS.^[^ ^146^ ^]^ The characterization indicated that Pt was slightly oxidized, and the valence states of the supports decreased, indicating electron transfer from Pt to the support. Furthermore, surface‐sensitive ultraviolet photoelectron spectroscopy (UPS) illustrates that the strong *p*–*d* orbital hybridization derived from electronic metal–support interactions can broaden the *d* levels of Pt and cause a narrower *d*‐band than that of bulk Pt materials. As a result, the electronic interaction with Pt–SAs/MoSe_2_ enhances intermediate affinity, leading to a boost in the HER. He et al. used DFT to calculate the electronic properties of single Pt atoms on CuO (Pt_1_–CuO).^[^ ^147^ ^]^ Calculations have shown that electrons migrated from Pt to CuO, resulting in an abundance of positively charged Pt atoms. The Pt–O–Cu coordination bonds also modulate the *d*‐band structure of Pt through electronic interactions. Pt_1_–CuO showed excellent catalytic performance toward acetone oxidation due to their lower energy barrier and facilitated rate‐limiting steps. Song et al. attribute the enhanced HER activity of Ru–Ni_5_P_4_ to electron redistribution and the formation of electronic metal–support interactions, as confirmed by XAS and DFT investigations.^[^ ^107^ ^]^ In summary, electron density redistribution is essential for enhancing performance by tuning the electronic structure of active atoms and regulating the reaction rate. The most important point is that the electronic metal–support interaction acts as a bridge between theoretical studies and practical experiments, providing instructive insights into explaining enhanced catalytic performance.

### 4.2 Chemical Covalent Bonding

Many compounds are stable in nature due to their covalent bonds and dispersed atoms in the SACs end, forming strong chemical bonds with surrounding coordination atoms, preventing agglomeration and ensuring good catalytic stability. Covalent bonding is the strongest metal–support interaction in SACs, also known as a covalent metal–support interaction, which is first proposed to explain the stability of Au_1_/FeO_*x*_ SAC.^[^ ^148^ ^]^ Some non‐C supports, such as metal oxides/hydroxides and metal‐derived compounds, are rich in coordinated atoms with Lewis base characteristics (lone electron pair and suitable electronegativity), enabling them to form stable covalent bonds with single metal atoms.^[^ ^149^ ^]^ Wang et al. reported the synthesis of uniformly dispersed Pt atoms on a CeO_2_ (Pt/CeO_2_) catalyst, in which a tight covalent Pt–O–Ce bond was formed via high‐temperature calcination.^[^ ^150^ ^]^ This bonding interaction not only stabilized the Pt single atoms but also improved the stability of the catalyst toward CO oxidation reactions. Similarly, Zhang et al. prepared Pt single atoms on FeO_*x*_ supports (Pt_1_/FeO_*x*_) via strong covalent Fe–O bonds on the surface,^[^ ^45^ ^]^ while Li et al. fabricated Pt single atoms with high loading amounts and uniform dispersions on MgAl_1.2_Fe_0.8_O_4_ spinel (Pt/MAFO) catalysts,^[^ ^151^ ^]^ which were attributed to the large surface area and the formation of covalent bonds in Pt/MAFO. Additionally, an ultrahigh‐loading Ir single atom (18 wt%) was successfully stabilized on the outermost surface of NiO via covalent Ir–O bonding.^[^ ^100^ ^]^ Moreover, other supports, such as sulfides and carbides, can also form the strong covalent bonds with single atoms. It is essential to note that the formation of covalent bonding interactions depends on the suitability of the substrate, and typical irreducible oxide supports, such as SiO_2_ and Al_2_O_3_, lack such interactions. In conclusion, the formation of covalent bonding interaction plays a critical role in enhancing both catalytic stability and formation stability, offering significant potential to address unstable catalytic properties and low loading amounts issues.

### 4.3 Synergistic Catalysis

The synergistic function is a term frequently used in catalysis, wherein two or more active centers can work together to conduct the catalysis. And synergy is one of the most advanced strategies for designing high‐performance catalysts by skillfully combining the advantages of different catalytic sites and making full use of the characteristics of each sites. Notably, in some non‐C‐supported SACs, the supports can also provide cooperative catalytic sites, further enhancing catalytic activity through significant metal–support interactions. The enhanced HER on the Pt single‐atom‐doped WO_3−*x*_ catalysts (Pt SA/WO_3−*x*_) was reported by Lee et al., which can be attributed to the hydrogen spillover effect at the interface.^[^ ^152^ ^]^ Specifically, H* is first formed at the Pt sites and then transferred to WO_3−*x*_ to produce H_*y*_WO_3−*x*_, wherein both Pt and H_*y*_WO_3−*x*_ synergistically act as active centers for H* combination and H_2_ generation, resulting in remarkable HER activity. Yamashita et al. prepared Ru single atoms loaded on H_*x*_MoO_3−*y*_, where Ru single atoms facilitate the activation and migration of H_2_, while Mo atoms are responsible for the absorption and dissociation of N_2_. These dual active sites of Ru and Mo jointly accelerate NH_3_ synthesis by a synergistic effect.^[^ ^153^ ^]^ Furthermore, Rh single atoms and the surrounding Pt atoms in Rh_at_O–Pt NCs act as absorption sites for ethanol, leading to the selective dissociation of the C–C bond and complete driving of EOR.^[^ ^86^ ^]^ Based on these examples, it is evident that non‐C‐supported SACs with well‐defined atomic and electronic structures can reasonably elaborate the synergistic function between reactive sites, unlike conventional heterogeneous materials (particles, clusters) with blurred active sites, and thus decipher the catalytic mechanism. In addition, the construction of dual‐atom catalysts with more active sites can further exploit the advantages of synergistic catalysis.

In summary, non‐C‐supported SACs have a wider variety of metal–support interactions than C‐supported SACs systems, making them more attractive. The electronic metal–support interaction plays a vital role in regulating the electronic structure, optimizing the catalytic pathway, and connecting with theoretical studies. Strong covalent bonding interactions between metals and non‐C supports increase the loading amount of single atoms, ensuring high stability and significant activity for the entire non‐C‐supported catalyst. Hence, a deeper understanding of the metal–support interaction is essential for deciphering the mechanisms, providing guidelines for preparing stable non‐C‐supported SACs, and modulating the catalytic performance.

## 5 Green Energy Conversion of Non‐C‐Supported SACs

Technologies for storing and converting renewable energy, such as fuel cells, metal–air batteries, and electrolyzers, are promising candidates for replacing conventional fossil fuels. The core of these environmentally friendly applications involves half‐reactions, including the HER, OER, ORR, ECR, and NRR, which can generate clean energy and valuable products. For enhancing the catalytic performance or product selectivity, electrocatalysts are indispensable in these reactions to depress the potential barrier, optimize the binding energy, and facilitate electronic interactions.^[^ ^7^, ^10^, ^11^ ^]^ SACs with the unique merits of maximum exposed active sites and unsaturated coordination are considered the most promising options compared to conventional catalysts. More attention has now been focused on the selection of non‐C materials for supporting single atoms to induce efficient metal–support interactions and electronic effects, further improving catalytic performance.^[^ ^59^, ^60^ ^]^ Hence, the recent progress in non‐C‐supported SACs in these representative reactions is discussed and concluded. In addition, the corresponding reaction mechanisms are presented through experimental and theoretical investigations, which offer a new direction for designing non‐C‐supported SACs with well‐defined structures for a variety of electrocatalytic reactions in the future.

### 5.1 HER

H_2_ is a promising energy carrier owing to its superior energy density and emission‐free output. Electrocatalytic water splitting is considered a valid strategy for generating H_2_ with high purity and zero emission.^[^ ^50^, ^154^, ^155^, ^156^ ^]^ Compared to C materials, non‐C materials used as supports for single atoms can effectively optimize the adsorption energy of H_2_ intermediates and induce distinct functions, such as hydrogen spillover, to accelerate the HER kinetics. Therefore, we have selected the representative non‐C‐supported SACs for HER and analyzed their promotion mechanisms and the relationship between their structure and performance. Additionally, **Table** **1** lists multiple non‐C‐supported SACs, along with their displayed catalytic activities and stabilities, providing a comprehensive review of recent research progress in HER catalysts.

**Table 1:** Summary of the HER performance for the non‐C‐supported SACs

Catalyst	Support	Overpotential	Tafel slope	Stability	Metal loading	Electrolyte	Electrode	Ref.
Co‐SAC/RuO_2_	RuO_2_	45 mV at 10 mA cm^−2^	58 mV dec^−1^	20 h at 10 mA cm^−2^	1.823 wt%	0.5 m H_2_SO_4_	Glassy carbon	[127]
Pt–V_2_CT_*x*_	V_2_CT_*x*_	27 mV at 10 mA cm^−2^	36.5 mV dec^−1^	10 h at 10 mA cm^−2^	0.88 wt%	0.5 m H_2_SO_4_	Glassy carbon	[190]
Mo_2_TiC_2_T_*x*_–Pt_SA_	Mo_2_TiC_2_T_*x*_	30 mV at 10 mA cm^−2^	30 mV dec^−1^	100 h at 10 mA cm^−2^	1.2 wt%	0.5 m H_2_SO_4_	Carbon paper	[157]
Pt/TiB_*x*_O_*y*_	TiB_*x*_O_*y*_	50 mV at 10 mA cm^−2^	32 mV dec^−1^	10 h at 10 mA cm^−2^	0.5 wt%	0.5 m H_2_SO_4_	Glassy carbon	[191]
Pt SA/WO_3−*x*_	WO_3−*x*_	47 mV at 10 mA cm^−2^	45 mV dec^−1^	–	0.42 wt%	0.5 m H_2_SO_4_	Glassy carbon	[152]
Pt/np‐Co_0.85_Se	np‐Co_0.85_Se	55 mV at 10 mA cm^−2^	35 mV dec^−1^	40 h at 10 mA cm^−2^	1.03 wt%	1 m PBS	Glassy carbon	[109]
SA In–Pt NWs	Pt	46 mV at 10 mA cm^−2^	32.4 mV dec^−1^	5 h at 10 mA cm^−2^	4.8 at%	1 m KOH	Glassy carbon	[90]
C‐Ir_1_/Co_0.8_Fe_0.2_Se_2_	Co_0.8_Fe_0.2_Se_2_	8 mV at 10 mA cm^−2^	–	–	2.0 wt%	1 m KOH	Glassy carbon	[138]
C‐Ir_1_/Co(OH)_2_	Co(OH)_2_	26 mV at 10 mA cm^−2^	–	–	2.0 wt%	1 m KOH	Glassy carbon	[138]
Ni_3_Fe–CO_3_^2−^ LDH–Pt SA	Ni_3_Fe–CO_3_^2−^ LDH	45 mV at 10 mA cm^−2^	37.8 mV dec^−1^	12 h at 10 mA cm^−2^	9.7 wt%	1 m KOH	Glassy carbon	[101]
Pt_SA_–NiO/Ni	NiO/Ni	26 mV at 10 mA cm^−2^	27.07 mV dec^−1^	30 h at 10 mA cm^−2^	1.14 wt%	1 m KOH	Carbon cloth	[192]
D‐NiO–Pt	D‐NiO	20 mV at 10 mA cm^−2^	31.1 mV dec^−1^	100 h at 10 mA cm^−2^	3.0 at%	1 m KOH	Carbon paper	[193]
Ru–a‐CoNi	a‐CoNi	15 mV at 10 mA cm^−2^	34 mV dec^−1^	–	5.35 at%	1 m KOH	Ni foam	[158]
Ru–c‐CoNi	c‐CoNi	56 mV at 10 mA cm^−2^	78 mV dec^−1^	–	5.76 at%	1 m KOH	Ni foam	[158]
Ru/np‐MoS_2_	np‐MoS_2_	30 mV at 10 mA cm^−2^	31 mV dec^−1^	40 h at 10 mA cm^−2^	8.0 at%	1 m KOH	Carbon cloth	[106]
Ni_5_P_4_–Ru	Ni_5_P_4_	54 mV at 10 mA cm^−2^	52 mV dec^−1^	10 k cycles	4.04 wt%	1 m KOH	Glassy carbon	[107]

**Figure 5:**
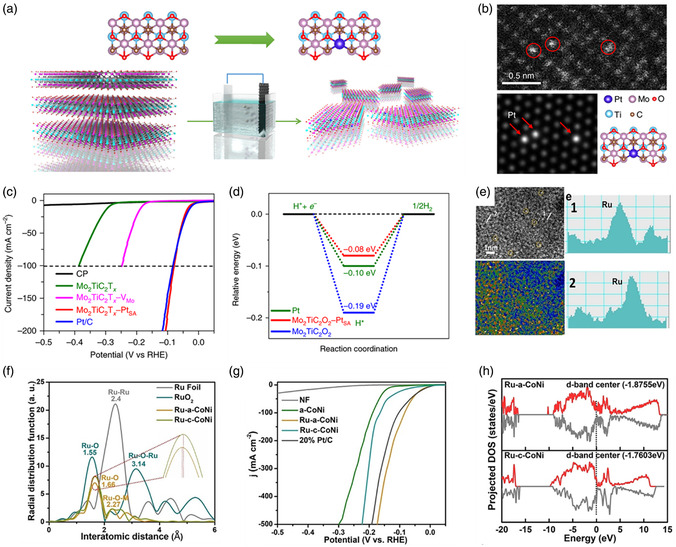
a) Schematic of the electrochemical exfoliation process of MXene with immobilized single Pt atoms. b) HAADF‐STEM image of Mo_2_TiC_2_T_*x*_–Pt_SA_ and its corresponding simulate image, and illustration of the structure of Mo_2_TiC_2_T_*x*_–Pt_SA_, showing the isolated Pt atoms. c) HER polarization curves of C paper (CP), Mo_2_TiC_2_T_*x*_, Mo_2_TiC_2_T_*x*_–V_Mo_, Mo_2_TiC_2_T_*x*_–Pt_SA_, and Pt/C (40%), acquired using graphite rod as the counter electrode in 0.5 m H_2_SO_4_ solution. d) Calculated free energy profiles of HER at the equilibrium potential for Mo_2_TiC_2_T_*x*_, Mo_2_TiC_2_T_*x*_–Pt_SA_, and Pt/C. a–d) Reproduced with permission.^[^ ^157^ ^]^ Copyright 2018, The Authors, published by Springer Nature. e) Atomic‐resolution STEM images and corresponding intensity profiles of Ru–a‐CoNi. f) EXAFS spectra of Ru in R‐space of all catalysts with standard references. Inset is the magnified view of the selected region. g) Polarization curves of HER of as‐obtained catalysts and h) the calculated PDOS of Ru–a‐CoNi and Ru–c‐CoNi. e–h) Reproduced with permission.^[^ ^158^ ^]^ Copyright 2022, Wiley‐VCH.

2D MXenes have great potential for various energy‐related applications due to their excellent metallic conductivity and chemical stability. They can also be used as supports with abundant coordinated environments for anchoring single metal atoms that exhibit excellent catalytic performance. For example, Wang et al. reported the synthesis of Pt single‐atom‐incorporated MXenes via electrochemical exfoliation, where abundant Mo vacancies (V_Mo_) were formed on the support surface.^[^ ^157^ ^]^ As shown in **Figure** **5**a, exfoliation and deposition were performed by conducting voltammetry scanning. Interestingly, during the process of applying voltage application, several Pt atoms were leached from the Pt foil and stabilized by isolated V_Mo_ via covalent bonding, producing the desired catalyst (Mo_2_TiC_2_T_*x*_–Pt_SA_) (Figure 5b). The HER performances were evaluated in 0.5 m H_2_SO_4_, and the corresponding polarization curves reveal that Mo_2_TiC_2_T_*x*_–Pt_SA_ with an extremely low Pt amount (1.2 wt%) shows Pt‐like HER activity and kinetics at 100 mA cm^−2^, which are markedly higher than those of Mo_2_TiC_2_T_*x*_ and Mo_2_TiC_2_T_*x*_–V_Mo_. Importantly, at an overpotential of 77 mV normalized to the Pt loading amount, the mass activity of Mo_2_TiC_2_T_*x*_–Pt_SA_ reaches 8.3 A mg^−1^, which is 39.5‐fold higher than that of a Pt‐on‐C catalyst. This indicates that maximum HER activity and economic efficiency can be realized on Mo_2_TiC_2_T_*x*_–Pt_SA_ (Figure 5c). Moreover, this catalyst exhibited excellent long‐term stability for 10 000 HER cycles or 100 h with negligible activity loss. Theoretical calculations illustrate that the enhanced HER activity on Mo_2_TiC_2_T_*x*_–Pt_SA_ is due to the relatively low H intermediate adsorption energy, which accelerates the formation and release of the generated H_2_ (Figure 5d). The strong covalent bonding interaction at the interface of Mo_2_TiC_2_T_*x*_–Pt_SA_ prevented surface diffusion and coarsening during the process, leading to the outstanding HER durability of Mo_2_TiC_2_T_*x*_–Pt_SA_. Lee et al. constructed single Ru atoms supported on an amorphous Co/Ni oxyhydroxide (Ru–a‐CoNi) catalyst via ambient NaBH_4_ reduction to unravel the metal–support interactions in the alkaline HER.^[^ ^158^ ^]^ For comparison, a crystalline counterpart (Ru–c‐CoNi) was prepared using a hydrothermal approach. As observed in the atomic‐resolution STEM image, isolated Ru atoms with higher brightness are marked in Figure 5e. The corresponding atomic intensity profiles further confirm the presence of single Ru atoms on c‐CoNi. The EXAFS results in Figure 5f illustrate the slightly shorter Ru–O peak in Ru–a‐CoNi than in Ru–c‐CoNi, indicating a closer metal–support interaction between Ru single atoms and the amorphous support, which is beneficial for electron transfer in Ru–O–Co/Ni moieties. Hence, Ru–a‐CoNi delivered an overpotential of only 15 mV in 1 m KOH, which was significantly lower than those observed for Ru–a‐CoNi and commercial Pt/C. Ru–a‐CoNi also possessed the best HER kinetics and stability among all the studied catalysts, further confirming its excellent synergistic function (Figure 5g). According to the mechanism discovered by DFT, it can be acknowledged that the d‐band position of Ru–a‐CoNi (−1.8755 eV) is more negative compared with the Ru–c‐CoNi (−1.7603 eV), which leads to the weakened affinity of H* intermediates (Figure 5h). Meanwhile, the possible d–d electrons transfer and medium‐to‐long rang p–*π* orbitals coupling induced on Ru–a‐CoNi could further intensify the metal–support interactions and enhance the HER performances.

In addition to the abovementioned samples, many other non‐C materials, such as MoS_2_ and WO_*x*_, are commonly used to construct high‐performance SACs for HER.^[^ ^106^, ^152^ ^]^ Notably, these non‐C materials usually have better corrosion resistance capability than carbon, resulting in longer stabilities of overall catalysts. The versatile electronic structures attributed to the induced metal–support interactions in such novel materials usually result in superior HER performance. Therefore, more effort should be made to explore non‐C‐supported SACs as promising HER catalysts.

### 5.2 OER

While the HER at the cathode is not a bottleneck, the OER at the anode presents a challenge due to its sluggish 4e^−^ transfer process, resulting in a high overpotential. Highly active and cost‐efficient SACs have emerged as promising electrocatalysts for the OER to overcome this issue.^[^ ^159^, ^160^ ^]^ Although most research has concentrated on accepting C as the support for SACs to conduct the OER in the last decades, it is easily dissolved under corrosive conditions and oxidative voltage, resulting in poor stability. Currently, an increasing number of studies are reported using non‐C materials, particularly metal oxides, to support isolated metal atoms that exhibit remarkable OER properties. This section introduces the recent progress in non‐C‐supported SACs, focusing on the function of the supports and the OER mechanism. The advanced non‐C‐supported SACs and their superior OER performances in different electrolytes are summarized in **Table** **2**.

**Table 2:** Summary of the OER performance for the non‐C‐supported SACs

Catalyst	Support	Overpotential	Tafel slope	Stability	Metal loading	Electrolyte	Electrode	Ref.
Ir–MnO_2_	MnO_2_	218 mV at 10 mA cm^−2^	59.61 mV dec^−1^	650 h at 10 mA cm^−2^	0.87 at%	0.5 m H_2_SO_4_	Glassy carbon	[163]
Ir–NiCo_2_O_4_	NiCo_2_O_4_	240 mV at 10 mA cm^−2^	60 mV dec^−1^	70 h at 10 mA cm^−2^	0.41 wt%	0.5 m H_2_SO_4_	Carbon cloth	[194]
Ru_1_–Pt_3_Cu	Pt_3_Cu	220 mV at 10 mA cm^−2^	–	28 h at 10 mA cm^−2^	8.2 wt%	0.1 m HClO_4_	Glassy carbon	[85]
Co–V_2_O_5_	V_2_O_5_	428 mV at 10 mA cm^−2^	92 mV dec^−1^	5 h at 10 mA cm^−2^	3.19 at%	0.1 m KOH	Glassy carbon	[128]
Ir_1_/Ni_1.6_Mn_1.4_O_4_	Ni_1.6_Mn_1.4_O_4_	390 mV at 100 mA cm^−2^	75 mV dec^−1^	60 h at 100 mA cm^−2^	0.459 wt%	0.5 m KOH + natural seawater	Ti net	[131]
Co‐SAC/RuO_2_	RuO_2_	200 mV at 10 mA cm^−2^	110 mV dec^−1^	20 h at 10 mA cm^−2^	1.823 wt%	1 m KOH	Glassy carbon	[127]
Ir_1_Co_13.3_O_20.1_	Co_13.3_O_20.1_	152 mV at 10 mA cm^−2^	60.5 mV dec^−1^	10 h at 10 mA cm^−2^	18.8 wt%	1 m KOH	Glassy carbon	[195]
Ir_1_/T_O_–CoOOH	T_O_–CoOOH	270 mV at 10 mA cm^−2^	65 mV dec^−1^	–	1.2 wt%	1 m KOH	Glassy carbon	[196]
Ir_1_/V_O_–CoOOH	V_O_–CoOOH	200 mV at 10 mA cm^−2^	32 mV dec^−1^	20 h at 10 mA cm^−2^	1.3 wt%	1 m KOH	Glassy carbon	[196]
A‐Ir_1_/Co_0.8_Fe_0.2_Se_2_	Co_0.8_Fe_0.2_Se_2_	235 mV at 10 mA cm^−2^	–	–	2.0 wt%	1 m KOH	Glassy carbon	[138]
A‐Ir_1_/Co(OH)_2_	Co(OH)_2_	260 mV at 10 mA cm^−2^	–	–	2.0 wt%	1 m KOH	Glassy carbon	[138]
Ir_1_/NFS	NFS	230 mV at 10 mA cm^−2^	36 mV dec^−1^	350 h at 100 mA cm^−2^	0.6 wt%	1 m KOH	Ni foam	[137]
Ir_1_/NFH	NFH	258 mV at 10 mA cm^−2^	50 mV dec^−1^	198 h at 100 mA cm^−2^	0.54 wt%	1 m KOH	Ni foam	[137]
Ir_0.1_/Ni_9_Fe	Ni_9_Fe	183 mV at 10 mA cm^−2^	49 mV dec^−1^	100 h at 10 mA cm^−2^	0.99 at%	1 m KOH	Ni foam	[162]
np‐Ir/NiFeO	NiFeO	197 mV at 10 mA cm^−2^	29.6 mV dec^−1^	80 h at 10 mA cm^−2^	0.1 wt%	1 m KOH	Ni foam	[162]
Ir_1_/Ni(OH)_2_	Ni(OH)_2_	260 mV at 10 mA cm^−2^	78 mV dec^−1^	260 h at 30 mA cm^−2^	5.81 wt%	1 m KOH	Glassy carbon	[102]
Ru/CoFe‐LDHs	CoFe‐LDHs	198 mV at 10 mA cm^−2^	39 mV dec^−1^	25 h at 200 mA cm^−2^	0.45 wt%	1 m KOH	Carbon paper	[134]
Ru–Co/LiCoO_2_	LiCoO_2_	247 mV at 10 mA cm^−2^	49.1 mV dec^−1^	190 h at 100 mA cm^−2^	5 wt%	1 m KOH	Carbon paper	[161]
Ru–NiCo_2_S_4–*x*_	NiCo_2_S_4–*x*_	190 mV at 50 mA cm^−2^	61.3 mV dec^−1^	12 h at 20 mA cm^−2^	2.46 at%	1 m KOH	Ni foam	[112]

**Figure 6:**
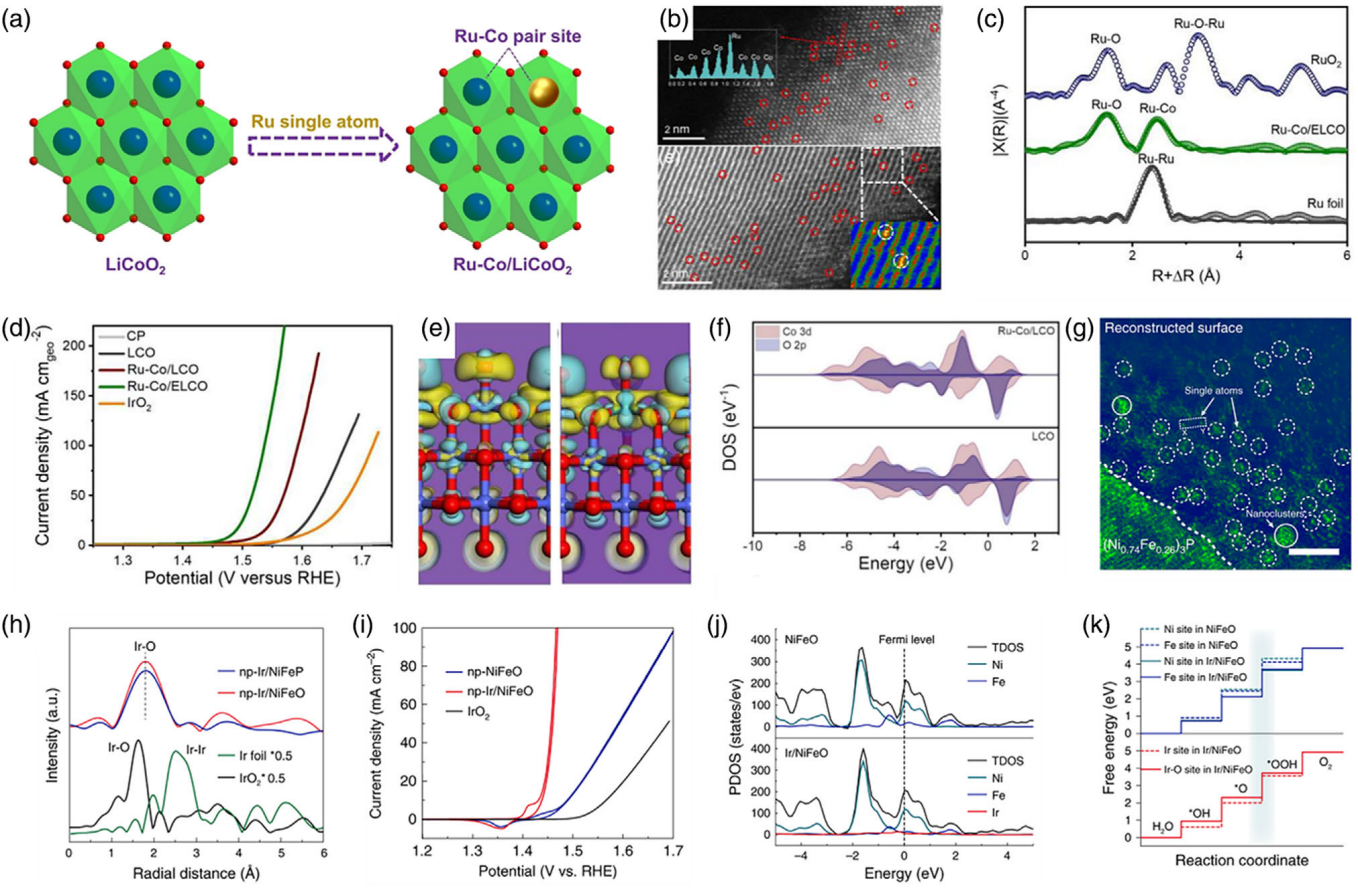
a) Schematic of Ru–Co/LCO SAC. b) HAADF‐STEM images with intensity profile and enlarged image for the selected rectangular area. c) The Ru K‐edge EXAFS spectra of Ru foil, RuO_2_, and Ru–Co/LCO SAC. d) The OER polarization curves of all catalysts. e) The charge density difference and f) PDOS of Co 3d and O 2p orbitals for LCO and Ru–Co/LCO SAC. a–f) Reproduced with permission.^[^ ^161^ ^]^ Copyright 2022, Wiley‐VCH. g) HAADF‐STEM image of np‐Ir/NiFeO, showing Ir atoms appear as bright spots and dispersed on the amorphous surface. h) EXAFS spectra of all catalysts. i) OER polarization curves of np‐NiFeO, np‐Ir/NiFeO, and IrO_2_. j) Calculated PDOS and k) calculated OER free energy diagram of NiFeO and Ir/NiFeO. g–k) Reproduced under the terms of the CC‐BY Creative Commons Attribution 4.0 International license (https://creativecommons.org/licenses/by/4.0).^[^ ^162^ ^]^ Copyright 2020, The Authors. Published by Springer Nature.

It is important to regulate the electronic properties and tune the energetics of the O_2_ intermediate adsorption/desorption of SACs by single‐site metal cation manipulation for the OER reactions. Li et al. recently reported a cation coordination method for constructing a Ru–Co/ELCO electrocatalyst with abundant Ru–Co pair sites by introducing single Ru atoms into a layer of LiCoO_2_ (ELCO).^[^ ^161^ ^]^ As revealed by the structural model in **Figure** **6**a, single Ru atoms substitute the Co atoms and are surrounded by six O atoms in Ru–Co/ELCO. Figure 6b clearly shows that atomically dispersed Ru atoms are present on the LCO support. The intensity profile and colored image further verify the substitution of Co atoms with Ru atoms in Ru–Co/ELCO. In addition, the Ru K‐edge EXAFS fitting results demonstrate that the coordination numbers of the Ru–O and Ru–Co bond are 6.3 and 5.6, respectively, which is consistent with the microscope results and strongly supports the close interactions between the Ru and Co atoms (Figure 6c). As shown in the OER polarization curves in Figure 6d, Ru–Co/ELCO displayed an overpotential of 247 mV, which was significantly lower than those of LCO (353 mV), Ru–Co/LCO (298 mV), and commercial IrO_2_ (361 mV). Notably, Ru–Co/ELCO displayed excellent stability for 190 h at a high applied current density, attributed to the good structural stability of the LCO support. From the charge density difference analysis by DFT calculations in Figure 6e, it is proven that the introduction of Ru atoms drives electron redistribution. In addition, the Co 3d and O 2p electron orbitals displayed more overlapping areas in Ru–Co/ELCO than in LCO, illustrating the strong electronic interactions in the Ru–Co pair sites (Figure 6f). These results illustrate that Ru‐single‐atom regulation can regulate the intrinsic Co electronic structure and boost the oxygen absorption kinetics, enhancing the OER activity. In contrast, surface self‐reconstruction induced by electro‐derived oxidation under OER conditions is a significant phenomenon when choosing non‐C materials, especially metal phosphides, as supports for SACs. However, scientists have found that structural changes may play an indispensable role in fixing single atoms and enhancing OER performance. Tan et al. employed a self‐reconstruction approach to synthesize high‐performance OER electrocatalysts with dispersed Ir atoms anchored to oxyhydroxides.^[^ ^162^ ^]^ Isolated Ir species were initially deposited on np‐NiFeP through electrochemical deposition in an electrolyte containing IrCl_3_ and KOH to obtain precatalysts (np‐Ir/NiFeP). Subsequently, the np‐Ir/NiFeP underwent further electrochemical activation, its surface was reconstructed, and the desired catalyst was generated (np‐Ir/NiFeO). As shown in Figure 6g, the typical HAADF‐STEM image indicates that the atomically dispersed Ir atoms are mainly located on the surface. Interestingly, the EXAFS spectra showed that the peak intensity of Ir–O in np‐Ir/NiFeO was higher than that in np‐Ir/NiFeP, suggesting that after surface reconstruction, the isolated Ir atoms were stabilized by more oxygen ligands (Figure 6h). Consequently, np‐Ir/NiFeO exhibits an ultralow overpotential of 197 mV in 1 m KOH. Normalized to the Ir loading, a 131‐fold enhancement in the mass activity was achieved for np‐Ir/NiFeO compared to that of IrO_2_ (Figure 6i). Additionally, the interactions between the more coordinated Ir atoms and the reconstructed surface augmented np‐Ir/NiFeO with excellent stability for almost 80 h. The results of the projected density of states (PDOS) calculations suggest a widening of the total density of states near the Fermi level due to the Ni and Fe atoms being optimized by the Ir atoms, resulting in a concentrated charge density on np‐Ir/NiFeO (Figure 6j). The OER free energy profile indicated that the rate‐determining energy barriers of the Ni and Fe atoms sharply decreased after the incorporation of single Ir atoms, indicating their OER‐favored kinetics and the formation of multiple active sites (Figure 6k).

Increasing research has been conducted, indicating remarkable performance toward the alkaline OER on non‐C‐supported SACs. The OER performed in an acidic electrolyte can provide a much higher H_2_ generation rate than that performed in an alkaline electrolyte and promoted the working efficiency of the water electrolyzer. However, only a few reports have used non‐C‐supported SACs as acidic OER electrocatalysts because most non‐C supports are more easily dissolved in acids. Researchers have recently found that some oxides, such as MnO_2_, can retain excellent OER stability under harsh conditions, such as oxidized voltage and strong acidity, and are promising candidates for supporting SACs.^[^ ^163^ ^]^ More effort is expected to be devoted to the design of non‐C‐supported SACs with excellent OER performances in acidic environments; however, this remains a great challenge.

### 5.3 ORR

The multielectron ORR can occur via a 4e^−^ process to produce H_2_O or via a 2e^−^process to produce H_2_O_2_. The 4e^−^ ORR is a significant reaction in proton‐exchange membrane fuel cells (PEMFCs), catalyzed by precious Pt to facilitate sluggish reaction kinetics. Considering their low cost, SACs are promising alternatives to Pt. Conversely, the 2e^−^ ORR usually requires isolated atoms to preserve the O–O bond to promote selectivity toward valuable H_2_O_2_, which means that SACs are the most suitable catalysts for this reaction.^[^ ^164^, ^165^, ^166^ ^]^ C‐supported SACs with good 4e^−^ ORR activity and high 2e^−^ ORR selectivity have already been investigated so far. Research on non‐C‐supported SAC with superior ORR performances is still not completely explored. Thus, we introduce representative ORR non‐C‐supported SACs in this section and expect to draw more attention to these novel catalysts. Emerging non‐C‐supported SACs with excellent ORR properties are listed in **Table** **3**.

**Table 3:** Summary of the 4e^−^ ORR and 2e^−^ ORR performance for the non‐C‐supported SACs

Catalyst for 4e^−^ ORR	Support	Activity	Half‐wave potential	Stability	Metal loading	Electrolyte	Electrode	Ref.
Co SA‐modified Pt NPs	Pt	0.77 mA cm^−2^ at 0.9 V	–	10 k cycles	0.9 wt%	0.5 m H_2_SO_4_	Glassy carbon	[80]
Pt_1_@Co/NC	Co	3.54 mA cm_Pt_^−2^ at 0.9 V	0.89 V	30 k cycles	2.2 wt%	0.1 m HClO_4_	Glassy carbon	[197]
Mo_1_/La_2_CoMnO_6_	La_2_CoMnO_6_	0.025 mA cm_OX_^−2^ at 0.99 V	–	20 h	0.06 wt%	0.1 m KOH	Glassy carbon	[167]
Pt_1_–Fe/Fe_2_O_3_	Fe_2_O_3_	54.2 mA cm_Pt_^−2^ at 0.9 V	1.05 V	50 k cycles	0.13 wt%	0.1 m KOH	Glassy carbon	[98]
Pt_quasi_/Mo_2_C	Mo_2_C	224 mA mg_Pt_^−1^ at 0.9 V	0.83 V	20 000 s	2.36 wt%	0.1 m KOH	Glassy carbon	[111]
Pt/MoN	MoN	710 mA mg_Pt_^−1^ at 0.9 V	–	30 000 s	0.2 wt%	0.1 m KOH	Glassy carbon	[103]
Pt/α‐MoC	α‐MoC	47.3 mA mg_Pt_^−1^ at 0.9 V	–	<30 000 s	0.2 wt%	0.1 m KOH	Glassy carbon	[103]
Catalyst for 2e^−^ ORR	Support	H_2_O_2_ selectivity	–	Stability	Metal loading	Electrolyte	Electrode	Ref.
Au_0.92_Pd_0.08_	Au	95% at 0 V	–	–	0.08 at%	0.1 m HClO_4_	Glassy carbon	[42]
h‐Pt_1_–CuS_*x*_	CuS_*x*_	96% at 0.7 V	–	10 k cycles	24.8 at%	0.1 m HClO_4_	Glassy carbon	[168]
Pt/TiN	TiN	65% at 0 V	–	–	0.35 wt%	0.1 m HClO_4_	Glassy carbon	[198]
Pt/TiC	TiC	71% at 0 V	–	–	0.2 wt%	0.1 m HClO_4_	Glassy carbon	[198]

**Figure 7:**
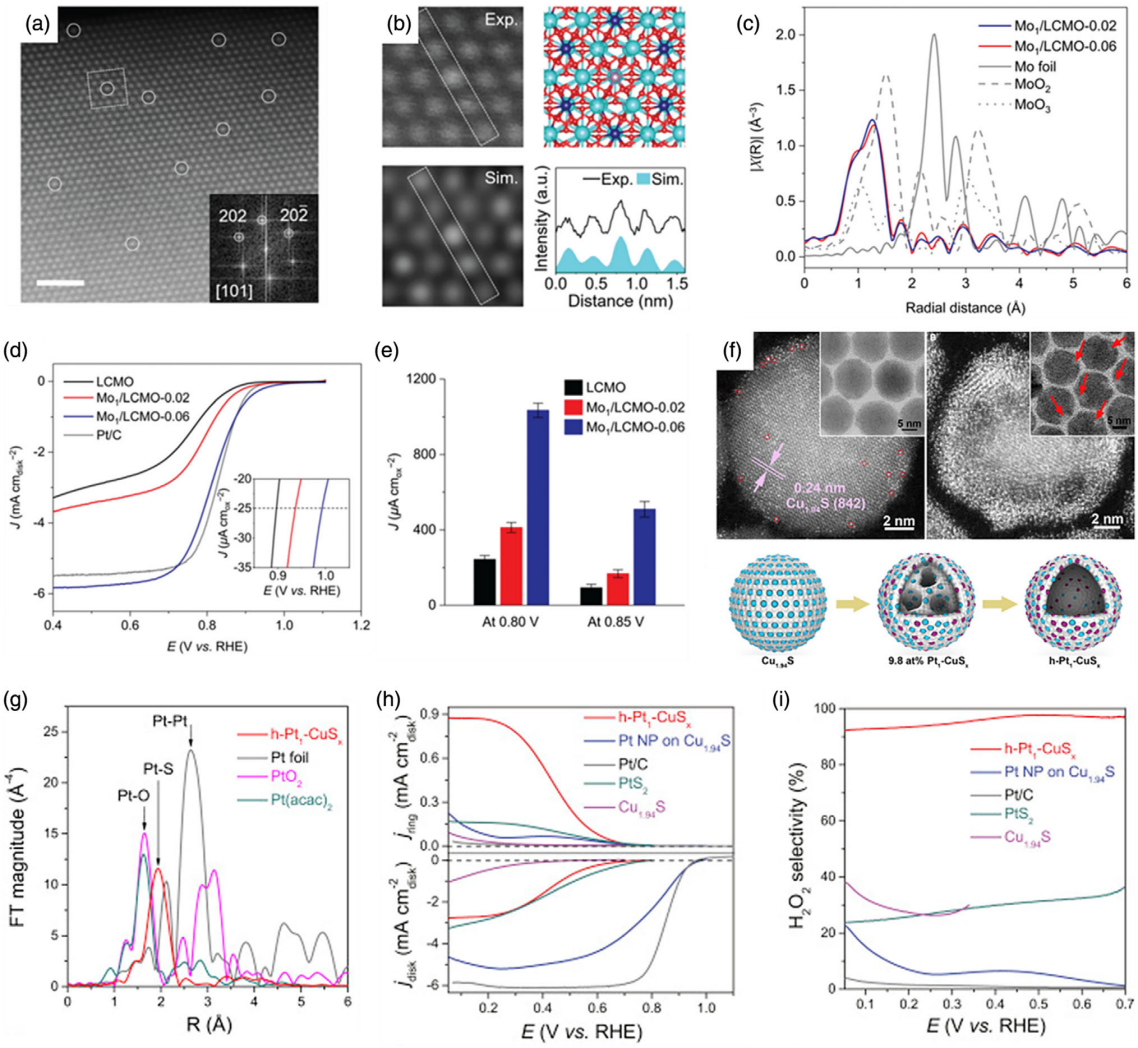
a) HAADF‐STEM image obtained along the [101] zone axis of Mo_1_/LCMO; the inset shows the FFT patterns. b) Enlarged view of the region marked in (a) and atomic model of Mo_1_/LCMO surface with its HAADF‐STEM image simulation and intensity profile analysis. c) EXAFS spectra of Mo_1_/LCMO and reference samples. d) ORR polarization curves of all catalysts and e) comparison of specific activities at 0.80 and 0.85 V versus RHE of LCMO and Mo_1_/LCMO. a–e) Reproduced with permission.^[^ ^167^ ^]^ Copyright 2021, Royal Society of Chemistry. f) AC HAADF‐STEM images of 0.68 at% Pt_1_–CuS_*x*_ (left) and 9.8 at% Pt_1_–CuS_*x*_ (right), and the schematic illustration of the structural evolution of h‐Pt_1_–CuS_*x*_. Blue, purple, and white balls represent Cu, Pt, and S atoms, respectively. g) Pt L_3_‐edge EXAFS profiles. h) RRDE measurement of the selective oxygen reduction in 0.1 m O_2_‐saturated HClO_4_ electrolytes and i) the H_2_O_2_ selectivity of these catalysts. f–i) Reproduced with permission.^[^ ^168^ ^]^ Copyright 2019, Elsevier Inc.

Metal oxides are frequently used in OER, but fewer studies have used them as electrocatalysts in the ORR due to their unsatisfactory activity. However, Li et al. reported the atomic incorporation of nonmagnetic hexavalent Mo (Mo^6+^) into the lattice of a perovskite oxide (La_2_CoMnO_6_) support (Mo_1_/LCMO) and showed enhanced ORR activity.^[^ ^167^ ^]^ As shown in **Figure** **7**a, Mo_1_/LCMO exhibited high crystal quality and no disorder, suggesting that Mo substitution did not deteriorate the lattice order. Some bright spots were distinguished as Mo atoms and occupied the B sites of the perovskite oxide. Figure 7b shows an enlarged view in which a single Mo atom with different contrasts is located at the center. The EXAFS spectrum without Mo–Mo scattering of Mo_1_/LCMO further indicates the isolated dispersion of Mo atoms (Figure 7c). As depicted in Figure 7d, the two Mo_1_/LCMO electrocatalysts show enhanced ORR activity compared to that of pristine LCMO. In particular, Mo_1_/LCMO–0.06 exhibited extremely high Pt‐like activity. Figure 7e further compares the specific activities under the applied voltages of 0.8 and 0.85 V, where the Mo_1_/LCMO–0.06 exhibits the highest ORR activity among all samples. Notably, the specific activity was 5 times higher than that of pristine LCMO at 0.8 V. Stability tests showed that the stability of Mo_1_/LCMO–0.06 decreases by 30% over 20 h of steady operation, whereas Pt/C displays a reduction of 30% in 6 h. Investigation of the mechanism revealed that the nonmagnetic Mo^6+^ in Mo_1_/LCMO–0.06 serves as an atomic‐scale electron trap, which can generate additional high‐spin, catalytically active Mn^3+^(t_2g_^3^eg^1^) sites, and highly conductive Co^2+^(eg^2^)–O–Mn^3+^(eg^1^) double exchange channels. DFT further confirmed a more exothermic reaction pathway and a lower barrier attributed to the doped Mo single atoms, leading to high ORR activity. This work illustrates that the single‐atom doping strategy has broad utility for inducing significant improvements in the ORR activity of metal oxides. In addition, the non‐C‐supported SACs showed remarkable H_2_O_2_ selectivity in the 2e^−^ ORR.^[^ ^168^ ^]^ For example, high‐loading Pt single atoms confined in hollow CuS_*x*_ supports (h‐Pt_1_–CuS_*x*_) were synthesized using an ion‐exchange approach. As shown in Figure 7f, Pt single atoms were first supported on the solid CuS_*x*_ (0.68 at% Pt_1_–CuS_*x*_) with a low concentration of H_2_PtCl_6_⋅6H_2_O and reaction rate. As the amount of Pt precursors increased and the reaction accelerated, the loading density of Pt single atoms increased, causing the formation of cavities on CuS_*x*_ (9.8% Pt_1_–CuS_*x*_). By increasing the reaction time further, the desired h‐Pt_1_–CuS_*x*_ was obtained with a hollow structure and a high Pt loading amount of 24.8%. The corresponding EXAFS spectra in Figure 7g reveal only one prominent peak assigned to the Pt–S bond in h‐Pt_1_–CuS_*x*_, indicating that the surrounding S coordination stabilizes isolated Pt atoms. The electrochemical activities of all the obtained samples for H_2_O_2_ production were determined in O_2_‐saturated HClO_4_ by rotating ring disk electrode (RRDE). As depicted in Figure 7h, h‐Pt_1_–CuS_*x*_ exhibited the highest current density, indicating its highest H_2_O_2_ selectivity (92–96%) compared to the other studied catalysts (<50%). The number of transferred electrons was approximately 2–2.2, suggesting a typical 2e^−^ ORR over h‐Pt_1_–CuS_*x*_ (Figure 7i). After 10 000 CV cycles, the H_2_O_2_ selectivity and current showed a limited loss for h‐Pt_1_–CuS_*x*_, which proves its good stability. The mechanism reveals that the conversion of adjacent Pt atoms into individual Pt atoms can effectively prevent the breaking of O–O bonds and inhibit the generation of O*, significantly enhancing H_2_O_2_ selectivity.

In short, novel non‐C‐supported SACs have shown promising potential for the ORR, especially the 2e^−^ pathway, which has high H_2_O_2_ selectivity. However, the stability issues of the electrocatalysts are negligible. For instance, the 2e^−^ ORR is commonly conducted in acid because H_2_O_2_ is unstable in alkaline solutions. This can be a limitation for the use of single atoms and supports efficiently. In the future, non‐C‐supported SACs with outstanding ORR activity, selectivity, and durability at various pH values can be expected to play an important role in the catalytic industry.

### 5.4 ECR

The multi‐C fuels and feedstock derived from ECR enable the reduction of greenhouse gas emissions and the sustainable utilization of CO_2_ and renewable electricity. To overcome the barrier for efficient ECR, it is important to choose electrocatalysts that activate the stable C–O bond in CO_2_ at room temperature and suppress the side reactions of the HER. C‐supported SACs have been extensively studied for ECR. However, most of the reported C‐supported SACs can only generate simple CO as their main product in the ECR. Currently, some non‐C‐supported SACs, especially SAAs with special electronic and geometric features, exhibit distinct ECR pathways and thus yield more valuable products.^[^ ^169^ ^]^ Therefore, two representative non‐C‐supported SACs for selectively reducing CO_2_ to formate and C_2_H_4_ are introduced in this section, with emphasis on their excellent ECR properties and reaction mechanisms.

**Figure 8:**
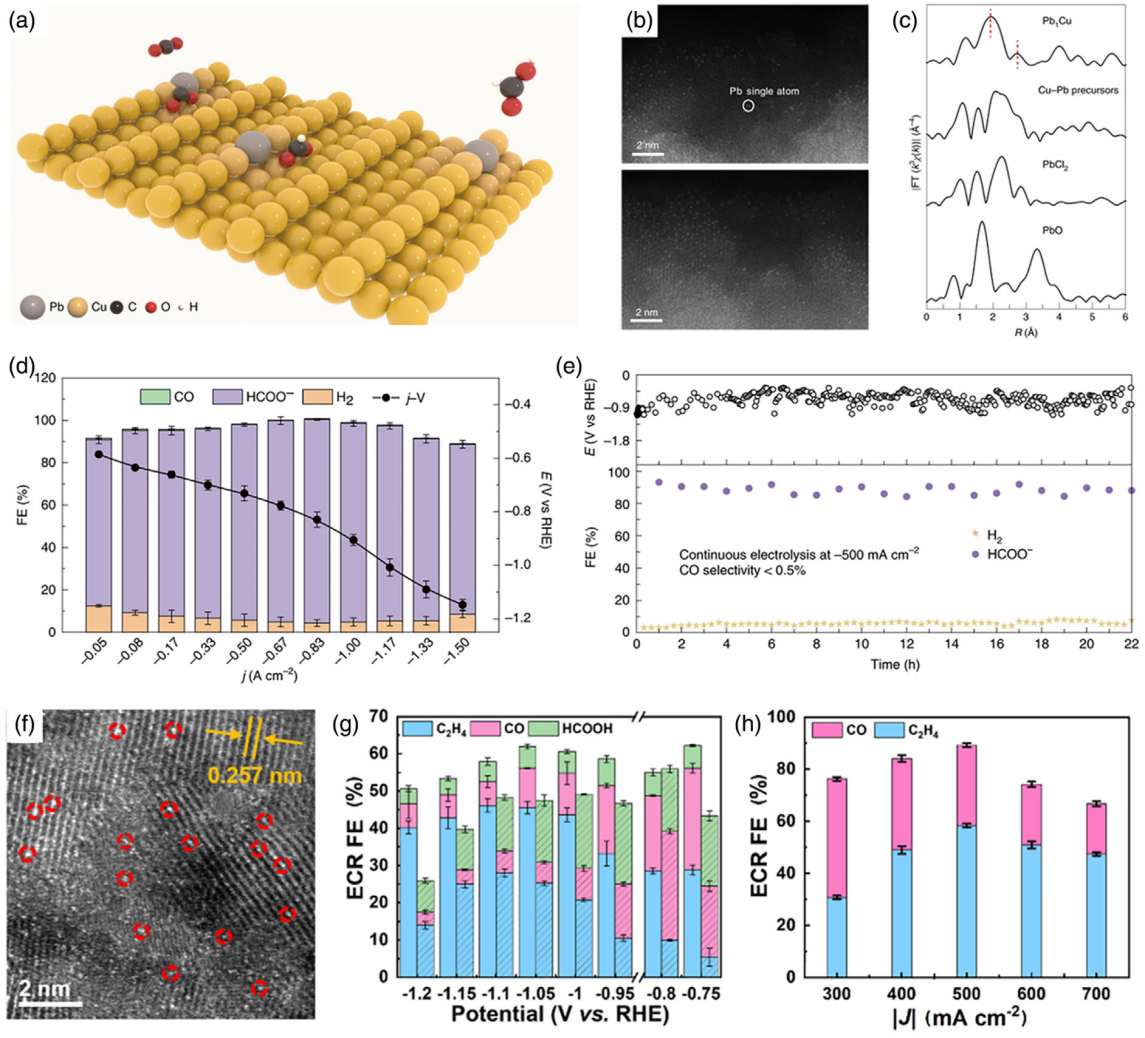
a) Schematic illustration of CO_2_ conversion into HCOOH over a Pb_1_Cu SAA. b) HAADF‐STEM images of the Pb_1_Cu catalyst. The white circle highlights the single‐dispersed Pb atom. c) Ex situ EXAFS spectra at the Pb L_3_‐edge of the Pb_1_Cu catalyst. d) FEs of all CO_2_RR products at different current densities and the corresponding *j*–*V* curves of the Pb_1_Cu catalyst. e) Stability test at −500 mA cm^−2^ current density in a flow cell for over 20 h, indicating an average FE of about 90%, estimated by NMR analysis. a–e) Reproduced with permission.^[^ ^170^ ^]^ Copyright 2021, The Authors, published by Springer Nature. f) HAADF‐STEM image of Sb/CuO (V_O_), some individual Sb atoms are marked with red dashed circles. g) Electrochemical CO_2_ reduction (ECR) FE over Sb/CuO (V_O_) (left) and CuO (V_O_) (right) against switching bias. h) FEs for ECR products at different applied current densities over single Sb/CuO (V_O_) in 1 m KOH electrolyte using a flow cell. f–h) Reproduced with permission.^[^ ^171^ ^]^ Copyright 2022, Wiley‐VCH.

As shown in **Figure** **8**a, Zeng et al. explored a Pb‐single‐atom‐alloyed Cu catalyst (Pb_1_Cu) for high‐performance ECR via an in situ electrochemical reduction method.^[^ ^170^ ^]^ Figure 8b shows the isolated dispersion of Pb atoms on a Cu support. In addition, Pb–Cu bonds were detected in EXAFS, verifying the strong metal–metal bond effect between the Pb and Cu atoms (Figure 8c). The ECR activity of Pb_1_Cu was evaluated using a three‐electrode flow cell containing 0.5 m KHCO_3_. As a result, it can be observed that the formate is the sole liquid product reduced by Pb_1_Cu. The largest formate FE of about 96% is reached with a partial current density of −800 mA cm^−2^ at −0.8 V. A high plateau of formate FEs was obtained over a wide range of potentials, whereas the competitive HER FE was completely suppressed. Notably, Pb_1_Cu showed a high partial current density of over −1000 mA cm^−2^ at approximately −1.0 V, while remarkable formate selectivity (92%) was maintained, outperforming many reported formate‐selective electrocatalysts (Figure 8d). In addition, the stability test was conducted at a current density of −500 mA cm^−2^, in which Pb_1_Cu can steadily operate for more than 20 h (Figure 8e). An investigation of the mechanism indicated that Cu species were the main sites for converting CO_2_ into formate. In situ spectroscopic evidence combined with DFT revealed that precise electronic and geometric adjustments using isolated Pb atoms on Cu could regulate the protonation step, divert the ECR toward the *OCHO pathway rather than the COOH* pathway, and increase the reaction barrier for the HER, resulting in excellent ECR properties. Moreover, multi‐C products such as C_2_H_4_ are more difficult to realize than single‐C compounds such as CO and formate because of the sluggish C–C coupling kinetics and slow multielectron transfer processes. To address these issues, Sun et al. coupled single Sb atoms and oxygen vacancies in CuO (Sb/CuO (V_o_)) for the first time to reduce CO_2_ to C_2_H_4_ at low overpotentials.^[^ ^171^ ^]^ As shown in Figure 8f, the HAADF‐STEM image shows the number of single Sb atoms on the CuO surface. ECR performances were evaluated in 0.1 m KHCO_3_, CO, HCOOH, and C_2_H_4_ are the reduction products on Sb/CuO (V_o_) within the switching potentials from −0.75 to −1.2 V. The transformation from CO_2_ to C_2_H_4_ is observed to occur at a low overpotential of less than 821 mV. C_2_H_4_ has gradually become the predominate ECR product when the potential is raised over −0.8 V, and the C_2_H_4_ FE exceeds over 40% in a wide potential range from −1.0 to −1.2 V on Sb/CuO (V_o_), which is much higher than those of CuO (V_o_) (Figure 8g). As shown in Figure 8h, although the current densities are largely scaled up, the total FE on Sb/CuO(V_o_) is still larger than 66% from 300 to 700 mA cm^−2^, and an impressive C_2_H_4_ FE of 58.4% is achieved at 500 mA cm^−2^. The excellent ECR properties and C_2_H_4_ selectivity of Sb/CuO (V_o_) are attributed to the increase in *CO coverage and low free energy of C–C coupling caused by the synergistic function between isolated Sb atoms and oxygen vacancies.

The induced metal–support interactions in non‐C‐supported SACs have been proven to effectively modulate the reaction barriers and pathways, and also suppress the HER, which can selectively generate multiple ECR products, including CO, formate, CH_4_, and C_2_H_4_.^[^ ^172^, ^173^, ^174^ ^]^ Although these initial achievements, efforts to continuously investigate emerging electrocatalysts for ECR should also be made in this field. We anticipate that the electronic and geometric functions and diverse active sites existing in non‐C‐supported SACs will drive the generation of sophisticated ECR products with increased C coupling in the future.

### 5.5 NRR

NH_3_ is a crucial cornerstone of the large and ever‐growing fertilizer and agricultural industries. Nowadays, industrial synthesis of NH_3_ mainly depends on the Haber–Bosch method, which consumes N_2_ and H_2_ at a high temperature of over 600 °C and pressure of over 40 MPa. Among the alternative methods, the NRR using water and N_2_ under mild conditions is considered a sustainable method for producing NH_3_. However, the main challenges in the NRR are the stable triple bonds in N_2_, which are very difficult to break, and the competing HER, which causes limited NH_3_ selectivity. Thus, it is crucial to explore efficient electrocatalysts for feasible NH_3_ production from inert N_2_ via the NRR. A variety of carbon‐supported Fe, Ru, Ni, Mo, and Cu SACs have been explored toward the NRR. In addition, non‐C‐supported SACs also show great potential for the NRR.^[^ ^175^ ^]^ For example, some metal oxide supports with easily formed vacancies are beneficial for N_2_ adsorption, and some metal supports can provide extra sites to work synergistically with single metal atoms to effectively dissociate the triple bond in N_2_, thereby improving the NRR FE.

**Figure 9:**
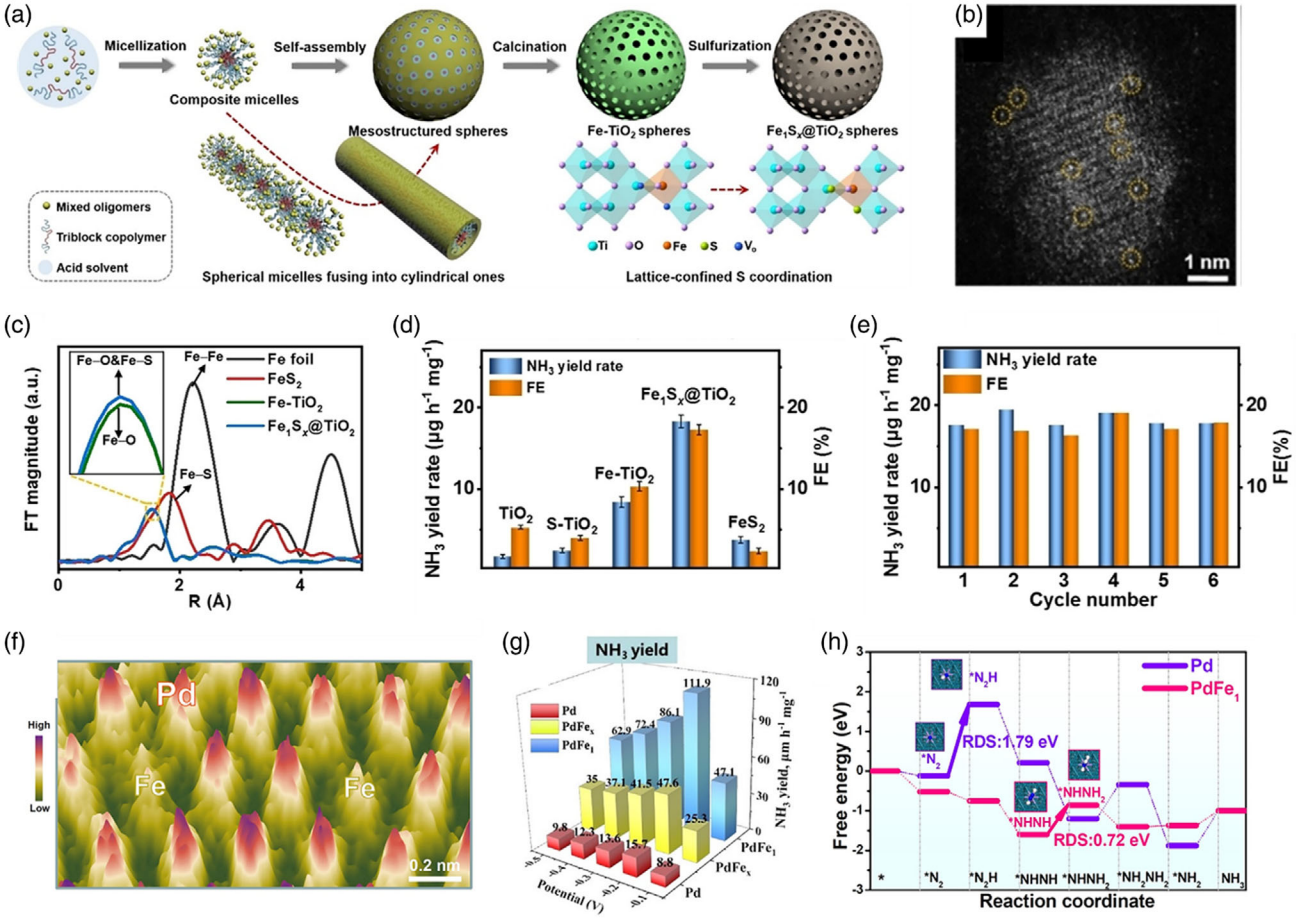
a) Schematic illustration of the synthesis of Fe_1_S_*x*_@TiO_2_. b) AC HAADF‐STEM image of the Fe_1_S_*x*_@TiO_2_ catalyst. c) The EXAFS spectra of the Fe R‐space for Fe_1_S_*x*_@TiO_2_, Fe–TiO_2_, FeS_2_, and Fe foil; inset is the magnification of the first peak. d) NH_3_ yield rate and FE, and e) NH_3_ yield rate, and FE in cycle tests at −0.2 V of Fe_1_S_*x*_@TiO_2_. a–e) Reproduced with permission.^[^ ^176^ ^]^ Copyright 2022, Wiley‐VCH. f) 3D topographic atom image of PdFe_1_. g) NH_3_ yields of Pd, PdFe_*x*_, and PdFe_1_. h) Free energy profiles of energetically preferred alternating NRR pathway on Pd and PdFe_1_ at zero applied energy. f–h) Reproduced with permission.^[^ ^88^ ^]^ Copyright 2022, Wiley‐VCH.

Li et al. reported the synthesis of S‐coordinated Fe SACs on TiO_2_ (Fe_1_S_*x*_@TiO_2_) for the electrocatalytic NRR.^[^ ^176^ ^]^ As shown in **Figure** **9**a, Fe_1_S_*x*_@TiO_2_ was prepared using a simple sulfurization strategy. HAADF‐STEM image indicated that the Fe single atoms are confined in the support lattice (Figure 9b). The Fe–S bond of Fe_1_S_*x*_@TiO_2_ in the EXAFS suggests an S‐decorated microenvironment of Fe single atoms (Figure 9c). The NRR activity of Fe_1_S_*x*_@TiO_2_ was determined in an H‐cell with 0.1 m HCl. At −0.2 V, Fe_1_S_*x*_@TiO_2_ obtains the fastest NH_3_ yield rate of 18.3 μg h^−1^ mg^−1^ and the highest FE of 17.3%, which are superior to other studied catalysts (Figure 9d). In addition, the NRR electrolysis was cycled 6 times with no obvious decay in the NH_3_ yield rate or FE, which confirms the excellent NRR stability of Fe_1_S_*x*_@TiO_2_ (Figure 9e). An investigation of the mechanism indicated that the mesoporous structure of TiO_2_ acts as a nanoreactor to accelerate mass transfer and increase the surface area. Moreover, S coordination regulates the local electronic structure of single Fe atoms in the TiO_2_ lattice, which can tremendously adsorb and activate N_2_, thus leading to an increased NRR. In addition, single Fe atoms confined on a Pd metallene support (PdFe_1_) were prepared by a hydrothermal approach and demonstrated to be a robust NRR catalyst.^[^ ^88^ ^]^ single Fe atoms were confirmed using 3D topographic atom imaging analysis (Figure 9f). The electrochemical NRR was performed in an electrochemical cell with 0.5 m LiClO_4_ solution. As a result, PdFe_1_ exhibits the highest NH_3_ yield rate and FE of 11.9 μg h^−1^ mg^−1^ and 37.8% at −0.2 V, respectively. In addition, an NRR stability test was performed on PdFe_1_, which showed a continuous current density and FE for 100 h. As revealed in Figure 9g, PdFe_1_ possesses the highest NRR activity at all potentials among the studied catalysts, where the optimized NH_3_ yield rate is 7.1 and 2.4 times higher than that of Pd and PdFe_*x*_, respectively, highlighting the significance of Fe single atoms. The corresponding mechanism disclosed by DFT in Figure 9h shows that Pd‐coordinated Fe single atoms can reduce the protonation energy barriers in the NRR and suppress the HER. Besides, the modulated electronic structure of single Fe atoms enables N_2_ activation via N_2_‐to‐Fe *σ*‐donation, which accounts for the superior NRR performances of PdFe_1_.

The yield rate of NH_3_ is still extremely low for all the explored electrocatalysts till now, making it difficult to meet the requirements of industrial applications.^[^ ^177^ ^]^ The development of NRR is still in its early stages, with many challenges, including the activation of inert N_2_ and suppression of the competing HER. However, recent advancements in non‐C‐supported SACs have shown promising performances toward NRR. We anticipate that this material system will address the aforementioned issues and promote NH_3_ production in the industry in the future.

## 6 In Situ/Operando Technologies for Non‐C‐Supported SACs

In the last decades, numerous sophisticated analytical tools have been successfully explored to obtain deep insights into the structure and electronic information of nanomaterials, to explore more in the field of material science. These traditional technologies can only characterize the catalyst before or after the reactions to deduce possible mechanisms. However, both the valence states and structures of the electrocatalysts undergo dynamic evolution, and some short‐lived intermediates are generated during electrocatalytic reactions. To directly monitor the dynamic process of an electrocatalyst under normal conditions, various in situ and operando methods, including STEM, XAS, infrared (IR) spectroscopy, and Raman spectroscopy, have been developed to obtain more accurate, in‐depth, and comprehensive evidence of catalysts during catalysis. These techniques are beneficial for deciphering the synthetic procedure, distinguishing the active sites, and elucidating the reaction mechanism.^[^ ^178^, ^179^ ^]^ Therefore, here, we summarize the advanced in situ/operando methods employed to study non‐C‐supported SACs, expecting that these successful applications will attract scientists to adopt these advanced tools to provide more insights in their work.

### 6.1 STEM

STEM is an important approach with sub‐Angstrom resolution for directly observing SACs, from which isolated metal atoms with different atomic numbers on the support can be clearly distinguished. However, the limited information provided by ex situ STEM can only reflect the external structures of SACs. It is vital to observe the atomic migration of SACs on the support during the synthetic process, which provide an evidence of the stabilization mechanism and offer a guideline for improving the synthetic protocols for SACs. With the rapid development of aberration correctors and monochromators, in situ/operando STEM has been exploited to directly monitor the dynamic evolution of specific regions of nanomaterials at the atomic scale under normal synthesis conditions, and has made progress in research regarding non‐C‐supported SACs.^[^ ^180^ ^]^

**Figure 10:**
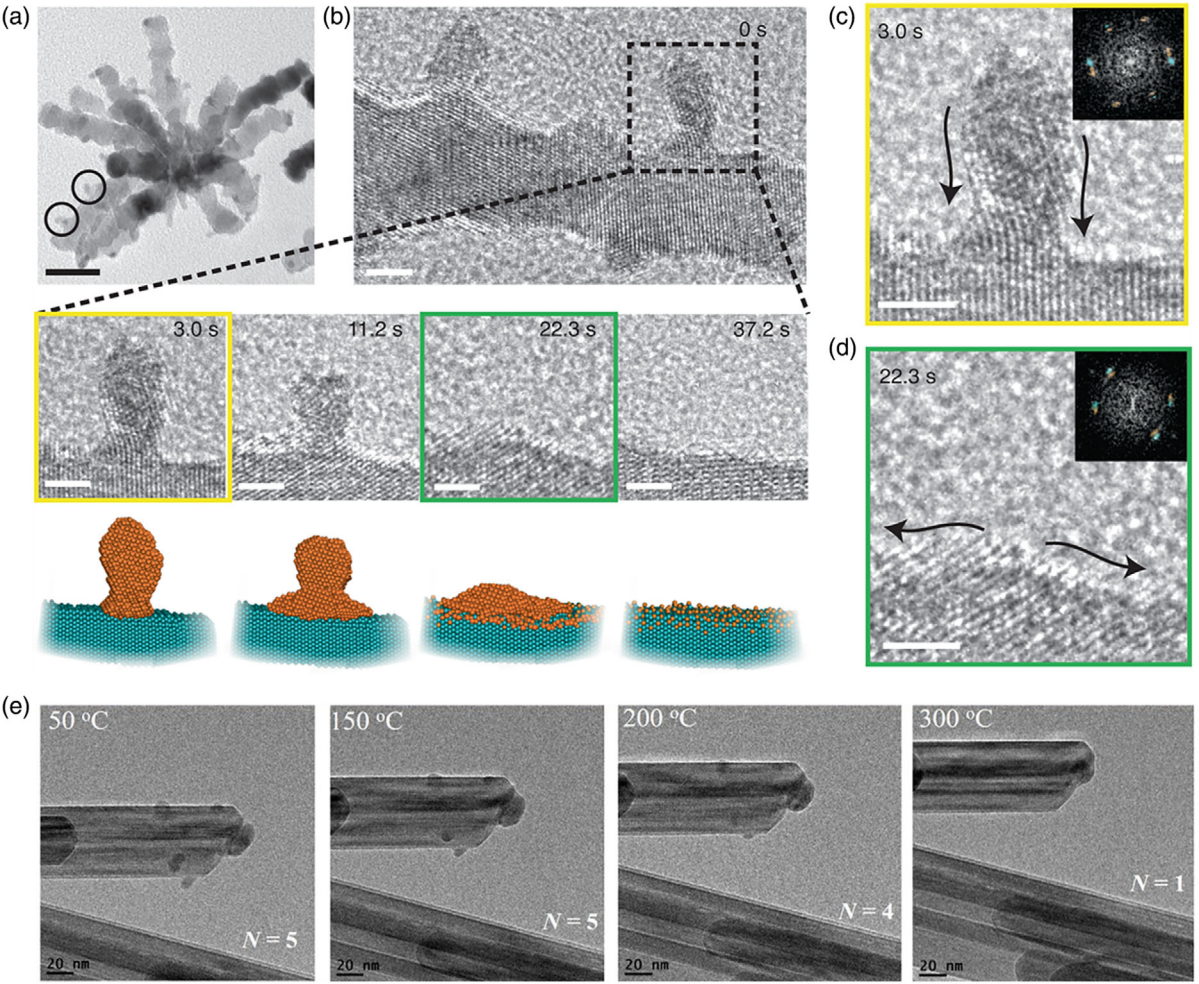
a) TEM image of Pt islands on a Ru branched nanoparticle with two typical Pt islands circled in black. Scale bar: 10 nm. b) HRTEM image of a Ru branch and Pt islands studied by in situ imaging, and HRTEM images and corresponding cartoons of the island in (b) as it transforms during in situ heating under a flow of H_2_. Scale bar: 2.5 nm. c,d) HRTEM images of the Pt branch after 3.0 s (c) and 22.3 s (d). The arrows illustrate the migration of Pt atoms. Insets: the corresponding FFTs of the images. a–d). Reproduced with permission.^[^ ^79^ ^]^ Copyright 2022, The Authors, published by Springer Nature. e) Representative TEM images acquired at different temperatures of Ag_1_/MnO_2_. Reproduced with permission.^[^ ^172^ ^]^ Copyright 2020, Wiley‐VCH.

Tilley et al. used in situ TEM to observe the formation of single Pt atoms on Ru nanoparticles.^[^ ^79^ ^]^ As shown in **Figure** **10**a, Pt islands with dimensions of 2.5 nm are grown. Then, the obtained nanomaterials were annealed in a H_2_/Ar gas flow, during which the Pt islands spread homogeneously over the Ru supports to form single Pt atoms. Atomic‐resolution images were obtained using in situ TEM to observe the dynamic evolution of the Pt structure over time to investigate the formation process. As a result, the small Pt islands remained stable during the initial 3.0 s of annealing. With increasing reaction time, the size of the Pt particles gradually decreased to 3.8 nm, indicating that the Pt atoms started to migrate on Ru. When the reaction time is increased to 22.3 s, the Pt atoms continue to spread. Finally, the Pt islands were completely dispersed as the isolated Pt atoms at the time of 37.2 s (Figure 10b). From the images produced by in situ TEM, the thermodynamic driving forces are summarized as the decreased surface energy of the Pt islands and the increased Pt–Ru bonds. The corresponding fast Fourier transforms (FFTs) collected at a reaction time of 3.0 s show hcp Ru and fcc Pt spots simultaneously (Figure 10c). However, only spots assigned to hcp Ru were observed at 22.3 s, indicating the formation of single Pt atoms rather than the Pt–Ru alloy (Figure 10d). Li et al. reported the thermal change of Ag nanoparticles to single atoms on MnO_2_.^[^ ^172^ ^]^ The in situ TEM was used to observe the atomization process to acquire more details of the conversion process (Figure 10e). Representative images were captured at different temperatures, where N is the number of remaining Ag nanoparticles in each image. The sizes of Ag nanoparticles show obvious decrease from 50 to 150 °C. When the temperature reaches 300 °C, diameters of both N and Ag nanoparticles significantly decline. Eventually, all Ag nanoparticles disappear on MnO_2_ supports at 350 °C and the generation of Ag single atoms was verified by HAADF‐STEM. Combined with other characterizations, the synthesis mechanisms were attributed to energetically favorable Ag single atoms and the trapping effect of O atoms in the MnO_2_ supports. In conclusion, in situ/operando STEM has successfully observed the real‐time transformation of nanoparticles to single atoms on supports, allowing for precise analysis of the driving force and formation mechanism. However, due to disturbances caused by the liquids, in situ STEM can rarely acquire high‐quality images in electrochemical environments or under wet‐chemistry synthesis conditions. Additionally, non‐C supports such as FeO_*x*_ are vulnerable to incident beams, making in situ STEM unsuitable for direct use with these materials. Therefore, a low‐loss imaging microscope technique with universality needs further development.

### 6.2 XAS

XAS is the most powerful and elaborate technique for determining the geometric chemistry and electronic structures of catalysts. XAS can be divided into XANES and EXAFS, based on the selected energy range. XANES can find out the information on the valence state, orbital occupancy, and site symmetry. Meanwhile, EXAFS revealed the coordination number, chemical bonds, and interatomic distances of the studied materials. It is also an indispensable method for ensuring the isolated atomic states of SACs based on chemical bond analysis. Owing to the rapid development of X‐rays with a deep penetration depth and high energy, in situ/operando XAS is now available for measuring dynamic changes in the chemical and electronic structures of catalysts during electrocatalysis while ignoring the influences caused by the electrolyte and atmosphere.^[^ ^181^, ^182^ ^]^ Recently, in situ/operando XAS has been widely employed in various electrocatalytic reactions to precisely distinguish the active sites and deduce their mechanism.^[^ ^183^ ^]^

**Figure 11:**
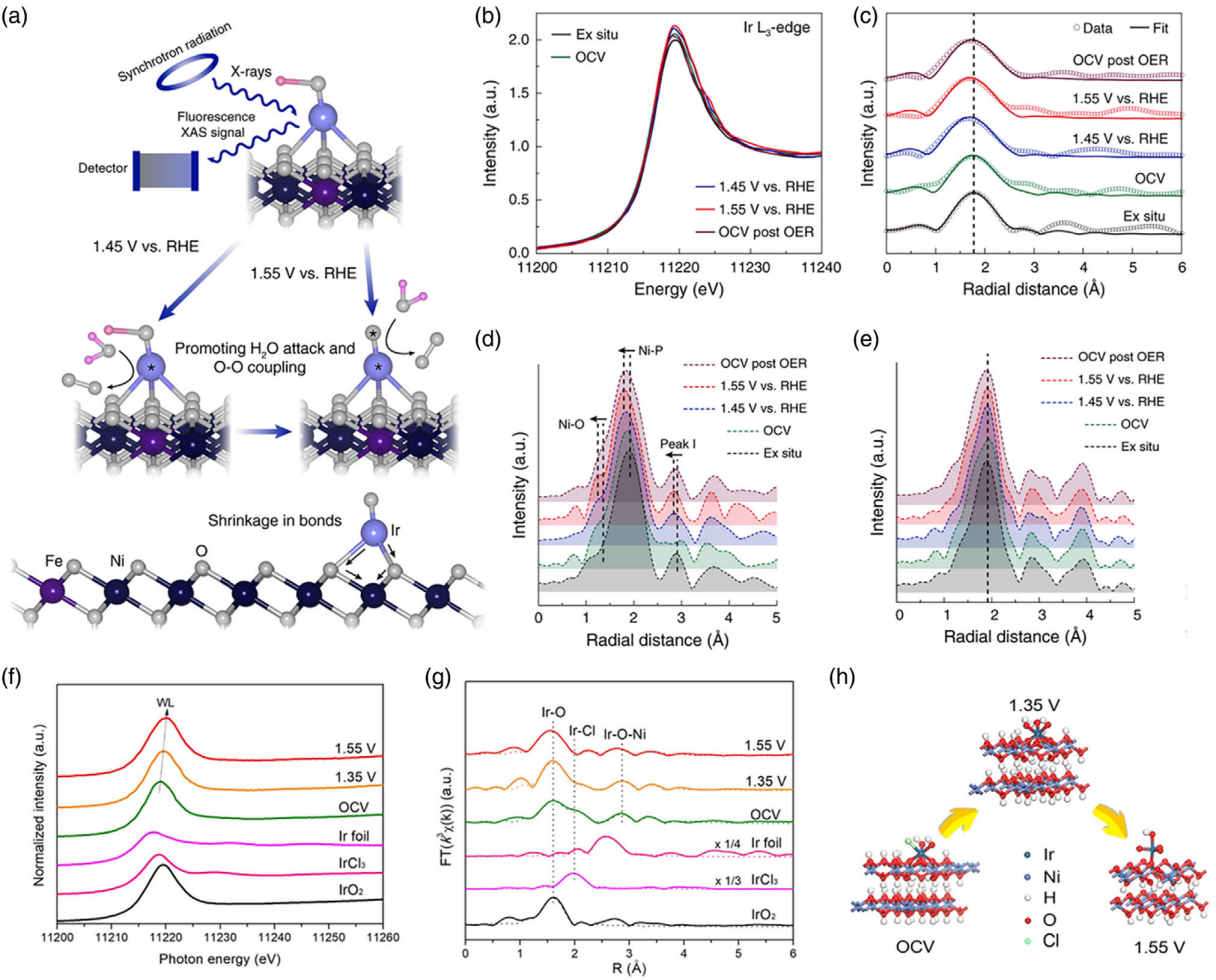
a) Schematic illustration of the OER mechanism determined by the operando XAS analysis of np‐Ir/NiFeO. b) Operando Ir L_3_‐edge XANES spectra of np‐Ir/NiFeO recorded from OCV to 1.55 V versus RHE in 1 m KOH. c) Corresponding first shell (IrO) fitting of FT‐EXAFS spectra for np‐Ir/NiFeO. d,e) Operando FT‐EXAFS spectra recorded at Ni K‐edge (d) and Fe K‐edge (e) under different applied voltages from OCV to 1.55 V versus RHE in 1 m KOH. a–e) Reproduced under the terms of the CC‐BY Creative Commons Attribution 4.0 International license (https://creativecommons.org/licenses/by/4.0).^[^ ^162^ ^]^ Copyright 2020, The Authors, published by Springer Nature. f) Ir L_3_‐edge XANES spectra measured at different potentials. g) Operando EXAFS fitting spectra collected at the Ir L_3_‐edge. h) Direct diagram of the electrochemical structure reconstruction process. f–h) Reproduced with permission.^[^ ^102^ ^]^ Copyright 2022, American Chemical Society.

Operando XAS was used to detect the real‐time changes in the valence states and local atomic environments that occurred during the reaction potentials in order to investigate the origin of the excellent catalytic performance as well as the structural advantages of np‐Ir/NiFeO (**Figure** **11**a).^[^ ^162^ ^]^ Normalized operando Ir L_3_‐edge XANES determined that the white line intensity increased at the open‐circuit voltage (OCV), which was caused by OH^−^ or H_2_O absorption. When the OER voltage was increased to 1.45 V, the white line intensity was higher than that at the OCV, indicating the formation of Ir intermediates and more absorbed OH^−^ or H_2_O (Figure 11b). These results are also supported by the operando Ir L_3_‐edge EXAFS, which shows a continuous increase of the Ir coordination number from ex situ condition to 1.45 V. Although the white line intensity of np‐Ir/NiFeO also shows a further increased state at 1.55 V, the corresponding EXAFS has no changes in comparison with that at 1.45 V, which suggests that the elevated valence state is not derived from the formation of Ir intermediates or absorption of oxygen‐containing species (Figure 11c). A deprotonation mechanism (Ir–OH to Ir–O*) at a higher applied OER potential is proposed, in which Ir displays a higher oxidation state without changing the coordination environment. Meanwhile, the oxygen species are activated in the process and function as synergistic sites to promote H_2_O attack and O–O coupling, facilitating kinetics. Moreover, the valence state of Ir can revert to its original state when the applied voltage returns to OCV, implying the good stability and reversible structure of this catalyst toward the OER. Interestingly, the operando Ni K‐edge EXAFS spectrum displays obvious negative shifts of all peaks in np‐Ir/NiFeO during the electrochemical OER, suggesting the contraction of Ni‐related bonds. The atomic environment of Ni could not revert to its original state, indicating the irreversibility of the Ni species during the OER (Figure 11d). Unlike Ni, the in situ Fe K‐edge EXAFS showed no change in electrocatalysis (Figure 11e). The enhanced OER mechanism of np‐Ir/NiFeO from the above XAS analysis is attributed to the fact that the Ir atoms with high valences and the activated oxygen species synergistically accelerate catalysis, and the stable surface of NiFeO further stabilizes the Ir single atoms. Song et al. employed operando XAS to investigate the OER catalyzed by Ir_1_–Ni(OH)_2_.^[^ ^102^ ^]^ Operando Ir L_3_‐edge EXAFS displays a steady increase in the valence states with increasing OER potentials (Figure 11f). An oxidation state of +5.5 was reached on Ir at 1.55 V; this phenomenon may be attributed to the absorbed O species. Notably, a peak assigned to the Ir–Cl bond appeared at the OCV and disappeared when the voltage increased to 1.35 V, indicating that the coordinated Cl was replaced by another O ligand. By further increasing the voltage to 1.55 V, the peak intensity of Ir–O–Ni was largely reduced, and small negative shifts were observed for both Ir–O and Ir–O–Ni, indicating dynamic reconstruction of the Ir sites and support (Figure 11g). According to the fitting results, the enhanced OER mechanism of Ir_1_–Ni(OH)_2_ was that the formation of high‐valence Ir efficiently promoted O–O formation (Figure 11h).

In brief, detailed evidence was provided on the real‐time changes in the electronic states and atomic microenvironments by applying in situ/operando XAS during catalysis. Such valuable information offers the opportunity to comprehend the role of elements, identify real active sites, and unravel the catalytic mechanism. Nevertheless, it should be emphasized that in situ/operando XAS is extremely sensitive to test devices and environments, particularly for SACs systems, and it is difficult to obtain fine data because of the low content of single atoms and the hindrance of the electrolyte to incident X‐rays. Moreover, the electrochemical reaction cells required for in situ/operando XAS must be customized for specific catalytic reactions and experimental facilities. Therefore, these rigorous conditions limit the application of in situ/operando XAS and urgent challenges must be resolved in the future.

Additionally, X‐ray photoelectron spectroscopy (XPS) and X‐ray diffraction (XRD) are two other techniques based on X‐rays. As for surface‐sensitive XPS, it is difficult to realize operando operation owing to the refraction of the electrolyte and the requirement of ultrahigh vacuum. A popular practice is quasioperando XPS, which characterizes a catalyst immediately after applying one voltage, followed by the application of the next voltage. For example, Zou et al. detected continuously increasing valence states of Pt and unchanged states of Fe in Pt/Fe_2_O_3_ during the ORR through quasioperando XPS, which indicated that OH* forms solely on Pt sites rather than Fe sites and distinguished the real active sites for ORR.^[^ ^98^ ^]^ The major limitation of quasioperando XPS is that it fails to monitor potential‐induced changes in catalysts under real‐time working conditions, whereas operando XPS development is still in progress. Operando XRD is a relatively mature tool for tracking phase transformations and crystallinities of catalysts. Chen et al. found that a new peak assigned to β‐MOOH emerges at 1.4 Å^−1^ when using operando XRD to observe the structural evolution of CoFe_2_O_4_ during OER, suggesting that the generated β‐MOOH is the OER active phase instead of γ‐MOOH analog.^[^ ^184^ ^]^ However, operando XRD may not be a universal tool for all samples, particularly for amorphous materials and SACs with no crystal phases.

### 6.3 IR Spectroscopy and Raman Spectroscopy

IR spectroscopy and Raman spectroscopy are basic analytical methods for determining the molecular structure of substances and identifying compounds based on the relative vibration and rotation between atoms within the molecules.^[^ ^178^, ^179^ ^]^ According to the limitations of the test conditions, Raman spectroscopy can be used to characterize substances that are inactive for IR; thus, the information contained in these two techniques is complementary in most cases. As the development of IR and Raman spectroscopy is mature, the equipment is simple, and the test process is facile and rapid, they have collaborated with reaction cells and upgraded as in situ/operando characterization methods. Raman spectroscopy is commonly more applicable and precise than traditional IR spectroscopy for electrocatalytic reactions because water has relatively low Raman scattering. Recently, IR with attenuated total reflection, which minimizes electrolyte interference and improves absorption signals, has been explored as a suitable mode for electrocatalytic studies. These two in situ/operando characterization methods are critical for detecting dynamically generated intermediates in the reaction process and have been successfully employed in many studies.^[^ ^181^, ^182^ ^]^

**Figure 12:**
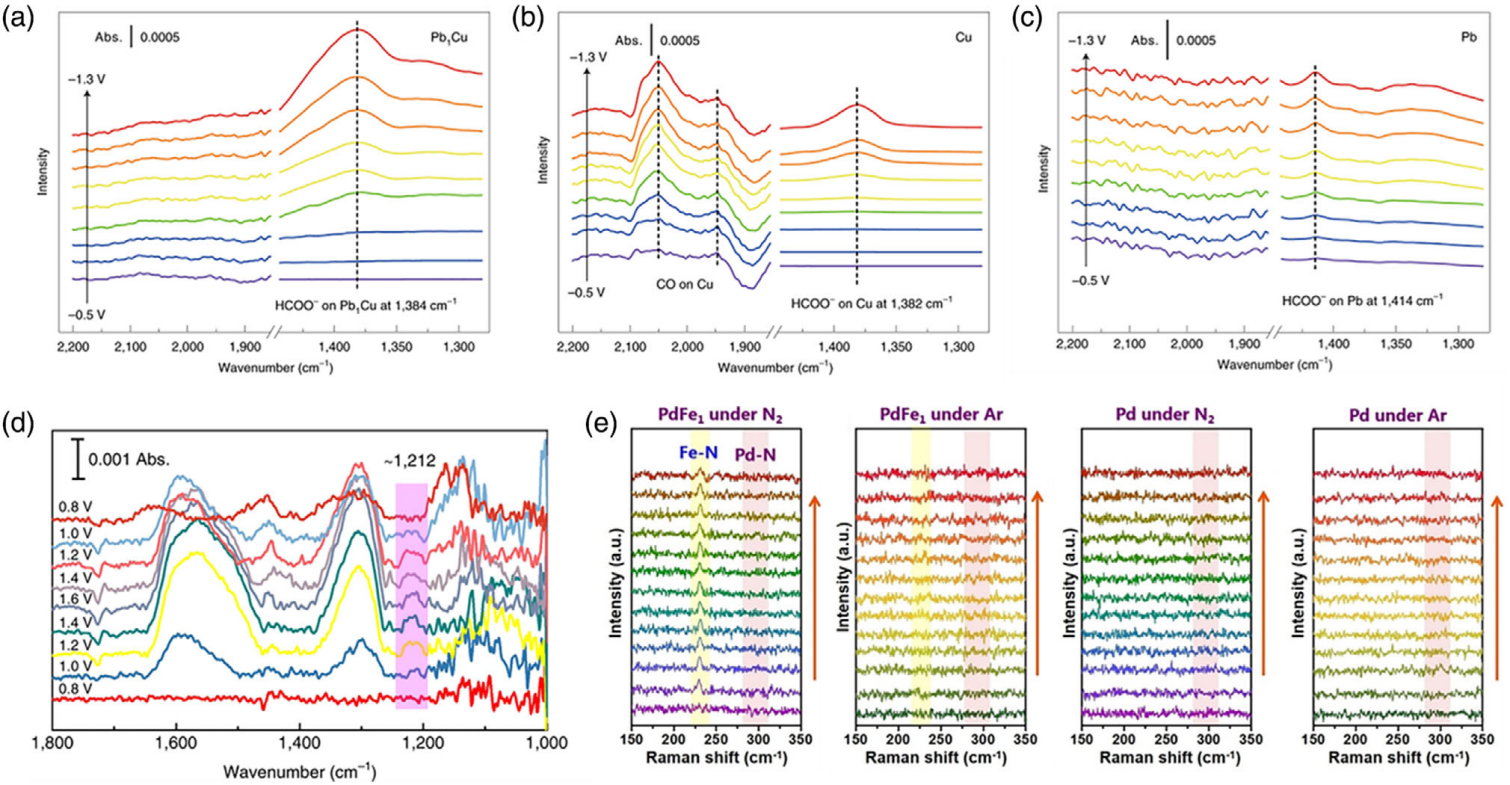
a–c) In situ ATR‐FTIR spectra recorded at different applied potentials for the Pb_1_Cu (a), Cu (b), and Pb (c) Pb. Reproduced with permission.^[^ ^170^ ^]^ Copyright 2021, The Authors, published by Springer Nature. d) In situ ATR‐IR spectra of Ru_1_–Pt_3_Cu recorded during the multipotential steps. Reproduced with permission.^[^ ^85^ ^]^ Copyright 2019, The Authors, published by Springer Nature. e) Operando Raman spectra of PdFe_1_ and Pd collected at different times of 0–60 min (−0.2 V) under N_2_/Ar conditions. Reproduced with permission.^[^ ^88^ ^]^ Copyright 2022, Wiley‐VCH.

For instance, to understand the ECR mechanism over Pb_1_Cu, Zeng et al. prepared pure Cu and Pb nanoparticles for comparison and employed in situ attenuated‐total‐reflectance Fourier transform infrared (ATR‐FTIR) spectroscopy to detect the reaction process on all samples.^[^ ^170^ ^]^ Two obvious IR bands appear at 1947 and 2050 cm^−1^ on the pure Cu from the applied voltages between −0.5 and −1.3 V, which are attributed to CO* derived from COOH* intermediates (**Figure** **12**b). In contrast, no IR band related to CO* appeared for Pb_1_Cu over the entire range of applied potentials, indicating a substantial C protonation mechanism instead of oxygen protonation. Additionally, peaks assigned to HCOO^−^ can be clearly observed on Pb_1_Cu and exhibit an increasing tendency of intensity, which confirms the growing formate production during ECR (Figure 12a). Moreover, the locations of HCOO^−^ IR bands on Pb_1_Cu, pure Cu, and Pb nanoparticles are 1384, 1382, and 1414 cm^−1^, respectively, suggesting that the intermediates have more tendency to absorb on Cu sites in Pb_1_Cu (Figure 12c). In light of these findings, the functions of the isolated Pb atoms were elucidated by modifying the geometric chemistry and electronic configurations of the Cu atoms, endowing Pb_1_Cu with a high current density and formate selectivity. In another case, Li et al. used the in situ ATR‐IR on Ru_1_–Pt_3_Cu to investigate the possible OER pathway.^[^ ^85^ ^]^ An evident peak at 1212 cm^−1^ belongs to the typical vibration of the absorbed OOH* can be clearly observed during OER working potentials (Figure 12d). Generally, the OOH* intermediate is considered the key evidence for the adsorbate evolution mechanism (AEM) in the OER. Additionally, the trend in the appearance of intermediates was consistent with the applied OER potentials, demonstrating a strong correlation between LOM and O_2_ production on Ru_1_–Pt_3_Cu. Operando Raman spectroscopy provided valuable information regarding the reaction intermediates. In order to get more insights of NRR on PdFe_1_, the time‐dependent operando Raman tests were conducted at −0.2 V in electrolytes containing N_2_ or Ar.^[^ ^88^ ^]^ Interestingly, a new stretching vibration peak assigned to Fe–N appears at 236 cm^−1^ and is maintained over the entire NRR process, whereas no Pd–N stretching vibration peak can be found at 285 cm^−1^, suggesting the valid N_2_ activation and dissociation on Fe single atoms. Similar to the findings for PdFe_1_, the Pd–N peak was also not detected on the pure Pd catalysts during the NRR, collectively demonstrating that the inactive Pd support has a negligible ability to absorb, activate, and dissociate N_2_, and the isolated Fe atoms are the real reactive sites toward the NRR.

Although in situ/operando IR and Raman spectroscopy have already been widely employed for detecting reaction intermediates, their application in SACs remains challenging due to the low content of metal atoms, which results in decreased signal intensity. In non‐C‐supported SACs, multiple adsorption sites may lead to interference if the signal positions are too close. Additionally, traditional IR and Raman spectroscopies with limited time response also restrict the detection of intermediates with short lifetimes (tens of seconds in general). Therefore, to expand the feasibility of in situ/operando IR and Raman technologies in the future, it is critical to adopt suitable electrochemical cells and sensitive detectors that can effectively enhance the temporal and spatial resolutions.

The research progress highlights that in situ/operando techniques are powerful tools; however, using only one characterization method can result in one‐sided and inaccurate information. The incorporation of multiple in situ/operando characterization tools, including microscopy and spectroscopy, is an imperative precondition for comprehensively resolving the geometric/electronic structures and comprehending the structure–property relationship. While some characterization methods have achieved breakthroughs in thermocatalysis, they are still rarely applied in the electrocatalytic field inevitable electrolyte interference, which presents an open area for further exploration. Moreover, a gap still exists between practical devices and in situ/operando electrochemical cells that are specially designed for characterization, making it difficult to simulate real operating conditions completely. Despite these current disadvantages, in situ/operando technologies remain indispensable strategies for obtaining detailed and in‐depth information about the structure and reaction mechanism of catalysts. These technologies will hopefully break the performance bottlenecks of electrocatalysts and drive their industrial applications forward in the coming decades.

## 7 Conclusions and Perspectives

Recent research has demonstrated the successful utilization of non‐C‐supported SACs in green energy conversion and their great potentials for further exploration. Non‐C supports can be divided into metals, metal oxides/hydroxides, metal‐derived compounds, and nonmetal compounds, all of which exhibit unique catalytic advantages. However, all synthesis methods have their advantages and disadvantages. Therefore, adopting an appropriate strategy is a vital prerequisite for preparing efficient non‐C‐supported SACs. Moreover, various metal–support interactions, including electron density redistribution, covalent bonding, and synergistic functions, are induced in non‐C‐supported SACs, which are indispensable for fixing single atoms and governing the catalytic performance. In order to prove the feasibility and reliability, these advanced non‐C‐supported SACs with different compositions and structures have been used in the electrochemical fields of HER, OER, ORR, ECR, and NRR, showing outstanding activity, stability, and selectivity. Owing to the progressive evolution of sophisticated in situ/operando technologies, the corresponding synthesis and reaction mechanisms have been elucidated. Some issues regarding non‐C‐supported SACs systems still need to be addressed before practical applications can be realized, as outlined below, along with perspectives on future development.

### 7.1 Preparation Issue

Many novel strategies have been explored for synthesizing non‐C‐supported SACs, of which bottom‐up methods such as impregnation are frequently used. Nevertheless, the loading amount of single metal atoms produced by these methods is much lower than that of the consumed precursor, leading to an increased cost. However, it is extremely difficult to realize mass production of non‐C‐supported SACs through bottom‐up methods. In contrast, a top‐down strategy can realize almost full metal utilization and large‐scale preparation per batch; however, harsh synthesis conditions are required. Therefore, more controllable and accessible approaches are required for non‐C‐supported SACs preparation in the future. In addition, high‐loading single atoms are a precondition for high performance in industrial‐scale applications; in particular, reactions with sluggish kinetics normally require more active sites to speed up the process, whereas the loading amount of currently reported SACs is still very low. It is desirable to construct supports with large surface areas and create abundant defects to provide more anchor sites to overcome this problem. In addition, the rational utilization of the covalent bonding interaction and spatial confinement effect is conducive for improving the loading amount of SACs. Importantly, achieving theory‐oriented synthesis of non‐C‐supported SACs with high performance is crucial in the future, and adopting DFT calculations is essential. DFT calculations not only shorten the unnecessary trial‐and‐error experimental process but also reduce cost, making them an important tool for achieving the desired performance of SACs.

### 7.2 Activity and Stability Issues

Although the electrochemical performances of the reported non‐C‐supported SACs have been significantly improved owing to their unique structures and efficient metal–support interactions, they are still far from satisfactory. Activity improvement is closely related to the microscopic structures of single metal atoms, which can be easily tailored by modulating the coordination environment of the supports. For example, Liu et al. constructed different cation vacancies (M^II^ or M^III^) and O atoms on a NiFe LDH to coordinate single Ru atoms.^[^ ^185^ ^]^ Consequently, Ru single atoms stabilized by M^III^ with Ru–O–Ni configurations possess the best catalytic performances among all studied catalysts, which is mainly due to the optimized absorption capability derived from the less populated *d* electrons on Ru. Thus, rational defect engineering on supports is an efficient method for adjusting the microenvironment of isolated atoms and optimizing the activity of non‐C‐supported SACs. In addition, the species of single atoms, which are normally transition metal elements with 3d to 5d orbitals, are important in catalysis. Recently, increasing attention has been paid to rare‐earth elements with inert 4f electrons, which can induce spin–orbital coupling. However, all rare‐earth SACs reported since 2018 are based on carbon, and the preparation of non‐C‐supported rare‐earth SACs and their electrocatalytic applications are promising fields that need to be explored.^[^ ^186^ ^]^ Moreover, diatomic catalysts (DACs) can provide more active sites and stronger synergies to boost catalytic reactions, but no research related to non‐C‐supported DACs has been conducted so far, which is worthy of the further investigation.^[^ ^187^ ^]^ Notably, the coexistence of single atoms and nanoclusters on non‐C supports may exhibit better catalytic performance than single atoms in some cases (nanoclusters will improve conductivity and charge transfer); thus, it is essential to regulate the proportion of single atoms and nanoclusters according to the actual situation and specific reaction.^[^ ^188^ ^]^ Although non‐C‐supported SACs are more stable than C‐supported SACs owing to stronger metal–support interactions, it is difficult to meet the requirements for practical applications (e.g., commercial PEM electrolyzers require at least 50 000 h of stable operation). The essential point is that catalytic reactions in industry are usually performed under harsh conditions with a higher current density and faster electrolyte flow compared with the measurements conducted in the laboratory. Such differences in the testing environment make it challenging for non‐C‐supported SACs to maintain the same high performance as that in the laboratory. Therefore, feasible non‐C‐supported SACs with more rigid structures and closer metal–support interactions must be exploited to retain high catalytic activity and durability in practical applications.

### 7.3 Elucidating Reaction Mechanism

On the one hand, in situ/operando techniques are undoubtedly the most cutting‐edge methods for investigating reaction mechanisms by monitoring the dynamic evolution of catalyst structures and generating intermediates during catalysis. However, for rapid electrocatalytic reactions in complex microenvironments, there still remain some shortcomings that need to be addressed. Initially, higher time resolutions of some microscopies and spectroscopies are required to match the rate of the reactions and capture the fleeting intermediates. In addition, the gap between in situ/operando testing devices and practical working equipment is anticipated to be bridged by simulating real electrochemical conditions. However, according to recent studies, the performance of SACs is influenced by the first coordinated shell, and second or even third atomic shell.^[^ ^189^ ^]^ The influence of higher shell atoms should not be ignored when speculating on the reaction mechanism for non‐C‐supported SACs with more variable moieties. Moreover, SACs usually maintain an unsaturated coordination to absorb reactants, whereas some ions such as K^+^ in electrolytes can also be absorbed at the same time. These inactive ions are supposed to adjust the electronic properties of the reactive single atoms; thus, the detailed functions of the absorbed ions in the catalytic process need to be determined, although they have still been overlooked. Furthermore, the significant function of DFT in disclosing catalytic mechanisms should be emphasized, as it opens a valuable pathway for monitoring electronic properties, calculating the absorption energy of intermediates, and determining the free energy of reactive steps. In particular, for SACs with simple and well‐defined configurations, DFT can establish more precise models in accordance with practical structures to aid in explaining the profound mechanism obtained experimentally.

## Conflict of Interest

The authors declare no conflict of interest.
